# Single-cell quantitative bioimaging of *P. berghei* liver stage translation

**DOI:** 10.1128/msphere.00544-23

**Published:** 2023-11-01

**Authors:** James L. McLellan, William Sausman, Ashley B. Reers, Evelien M. Bunnik, Kirsten K. Hanson

**Affiliations:** 1 Department of Molecular Microbiology and Immunology and South Texas Center for Emerging Infectious Diseases, University of Texas at San Antonio, San Antonio, Texas, USA; 2 Department of Microbiology, Immunology, and Molecular Genetics, Long School of Medicine, University of Texas Health Science Center, San Antonio, Texas, USA; University at Buffalo, Buffalo, New York, USA

**Keywords:** malaria, drug discovery, quantitative bio-imaging, liver stage

## Abstract

*Plasmodium* parasite resistance to existing antimalarial drugs poses a devastating threat to the lives of many who depend on their efficacy. New antimalarial drugs and novel drug targets are in critical need, along with novel assays to accelerate their identification. Given the essentiality of protein synthesis throughout the complex parasite lifecycle, translation inhibitors are a promising drug class, capable of targeting the disease-causing blood stage of infection, as well as the asymptomatic liver stage, a crucial target for prophylaxis. To identify compounds capable of inhibiting liver stage parasite translation, we developed an assay to visualize and quantify translation in the *Plasmodium berghei-*HepG2 infection model. After labeling infected monolayers with o-propargyl puromycin (OPP), a functionalized analog of puromycin permitting subsequent bioorthogonal addition of a fluorophore to each OPP-terminated nascent polypeptide, we use automated confocal feedback microscopy followed by batch image segmentation and feature extraction to visualize and quantify the nascent proteome in individual *P. berghei* liver stage parasites and host cells simultaneously. After validation, we demonstrate specific, concentration-dependent liver stage translation inhibition by both parasite-selective and pan-eukaryotic active compounds and further show that acute pre-treatment and competition modes of the OPP assay can distinguish between known direct translation inhibitors and other compounds which we identify as indirect translation inhibitors. Using this framework, we identify a Malaria Box compound, MMV019266, as a direct translation inhibitor in *P. berghei* liver stages and confirm this potential mode of action in *Plasmodium falciparum* asexual blood stages.

**IMPORTANCE:**

*Plasmodium* parasites cause malaria in humans. New multistage active antimalarial drugs are needed, and a promising class of drugs targets the core cellular process of translation, which has many potential molecular targets. During the obligate liver stage, *Plasmodium* parasites grow in metabolically active hepatocytes, making it challenging to study core cellular processes common to both host cells and parasites, as the signal from the host typically overwhelms that of the parasite. Here, we present and validate a flexible assay to quantify *Plasmodium* liver stage translation using a technique to fluorescently label the newly synthesized proteins of both host and parasite followed by computational separation of their respective nascent proteomes in confocal image sets. We use the assay to determine whether a test set of known compounds are direct or indirect liver stage translation inhibitors and show that the assay can also predict the mode of action for novel antimalarial compounds.

## INTRODUCTION

*Plasmodium* parasites are the causative agent of malaria and continue to have an outsized effect on global public health, causing an estimated 241 million cases in 2020, with 77% of deaths occurring in children under the age of 5 (1). Antimalarial drugs are essential for treating malaria; however, all currently used antimalarials are associated with parasite resistance. The spread of *kelch13-*mediated resistance to the antimalarial artemisinin in Southeast Asia and its recent *de novo* emergence in Rwanda demonstrates the critical threats to the efficacy of artemisinin combination therapies, the front-line therapeutics targeting asexual blood stage (ABS) parasites, which cause all malaria symptoms (2–5). In addition to treating malaria, antimalarial drugs would ideally be able to clear any non-replicative gametocytes in the blood, preventing transmission back to the mosquito vector. Antimalarials are also crucial for disease prophylaxis, with the *Plasmodium* liver stage (LS) a key target to prevent both disease and transmission (6). Attractive antimalarials would thus have activity against each of these three stages despite significant stage-specific differences in biology (7–9), highlighting the utility of targeting core cellular processes, like translation, that are crucial for all mammalian stages of development.

Translation of mRNA nucleotide sequences to amino acids during the ribosomal synthesis of proteins is a central evolutionarily conserved cellular process that has been extensively targeted with antibiotics treating bacterial infections (10), but *Plasmodium* translation has not been targeted by any clinically approved antimalarials to date. *Plasmodium* protein synthesis is a highly desirable process to target, as translation can be blocked via many different molecular targets. DDD107498 (also known as cabamiquine and M5717), which is thought to target eEF2, a core component of polypeptide elongation on the ribosome (11), and a number of cytoplasmic aminoacyl-tRNA synthetase inhibitors, which prevent the linkage between a tRNA and its cognate amino acid, are in various stages of clinical and pre-clinical development, respectively (12, 13). Additionally, many pan-eukaryotic translation inhibitors have antiplasmodial activity against *Plasmodium falciparum* ABS in standard 48-hour (h) assays and were shown to directly target the cytoplasmic translation apparatus using a bulk ABS lysate approach in which translation of the exogenous luciferase transcript is used as a biomarker for total cellular translation (14, 15). Currently, the ability to gain such mechanistic information about antimalarial activity is almost entirely dependent on ABS experiments (16), with the assumption that antiparasitic activity in other stages occurs via the same mechanism. LSs are particularly problematic as they rely on highly metabolically active hepatocytes for their own development, which makes bulk population readout of conserved processes like translation impossible due to the signal from hepatocytes themselves. It also complicates the interpretation of LS antiplasmodial activity, as it may integrate both hepatocyte- and parasite-directed effects.

Here, we report a bioimage-based assay quantifying *Plasmodium berghei* LS translation in the native cellular context. We rely on the activity of the aminoacyl-tRNA mimic puromycin, which is covalently bound to the C-terminus of a nascent polypeptide during the elongation reaction, causing the ribosome to disassociate and release the puromycin-bound nascent-polypeptide (17–21). A synthetic puromycin analog, o-propargyl puromycin (OPP), was shown to truncate and label nascent polypeptides in an identical manner but contains a small alkyne tag, facilitating the copper-catalyzed cycloaddition of a picolyl azide fluorophore in a bioorthogonal reaction, commonly termed “click chemistry” (22). Combining the OPP labeling of nascent polypeptides with automated fluorescence microscopy and quantitative image analysis, we demonstrate specific and separable *in cellulo* quantification of *P. berghei* and *Homo sapiens* translation during LS development in HepG2 cells and use the assay to identify both direct and indirect inhibitors of *Plasmodium* LS translation.

## RESULTS

### Visualization of the *Plasmodium* nascent proteome

**Fig 1:**
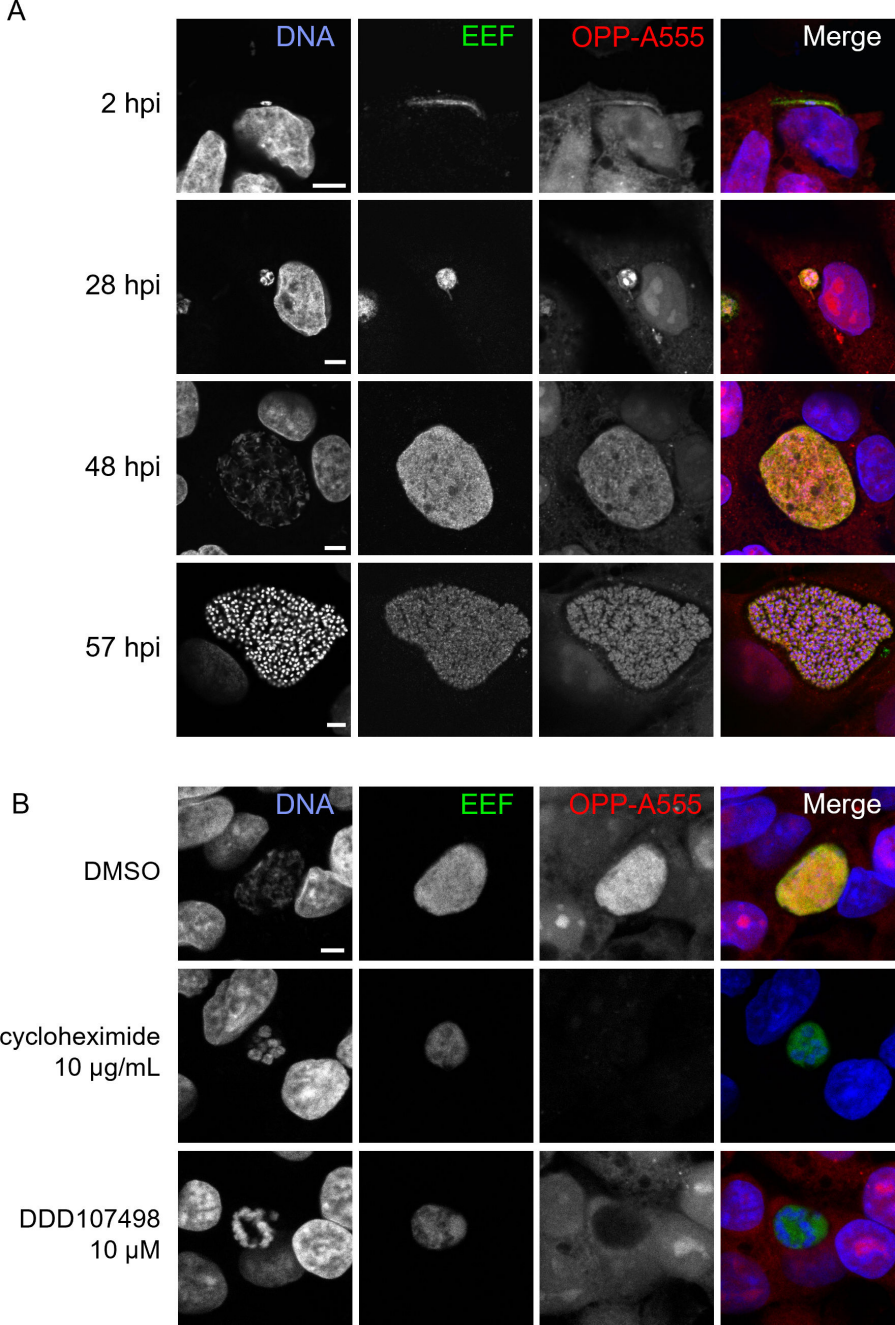
Visualization of the nascent proteome in *Plasmodium berghei* LS parasites. (**A and B**) Representative, single confocal images of *P. berghei-*infected HepG2 cells, with OPP conjugated to Alexa Fluor 555 (OPP-A555) labeling the nascent proteome in both HepG2 and parasite [exoerythrocytic parasite forms (EEF)], with Hoechst labeling DNA. Single channel images are shown in grayscale, with merges pseudo colored as labeled. (**A**) Visualization of the nascent proteome throughout LS development, with parasite immunolabeled with α-UIS4 (2 hpi) or α-HSP70 (28, 48, and 57 hpi). (**B**) Nascent proteome visualized in infected HepG2 cells following treatment from 44 to 48 hpi with translation inhibitors cycloheximide (10 µg/mL), or DDD107498 (10 µM) vs dimethyl sulfoxide (DMSO) control. All images in B were acquired and processed with identical settings and are shown at the same scale; scale bars = 5 µm.

With a goal of quantifying translation in single parasites, we first explored whether OPP would label the *P. berghei* nascent proteome during LS development. Infected HepG2 cells were treated with OPP for 30 minutes at 37°C, then immediately fixed with 4% paraformaldehyde, which stops the labeling reaction and preserves the quantity and cellular localization of the OPP-labeled polypeptides (22). Post-fixation, a click chemistry reaction attaches a picolyl azide conjugated fluorophore to the OPP-labeled polypeptides of both host and parasite (Fig. S1A), which can then be visualized with fluorescence microscopy. AlexaFluor555 was used to visualize newly synthesized peptides throughout this study, and the resulting signal, from both host and parasite nascent proteomes, will be referred to as OPP-A555. As expected, we can visualize translation throughout LS parasite development (Fig. 1A), from newly invaded sporozoite (2 hpi), through merozoite formation (57 hpi). By eye, parasite translation intensity (evidenced by OPP-A555 signal) appears generally greater than that of the host cell and surrounding non-infected HepG2 cells. The robust and highly specific OPP-A555 signal (Fig. S1B) suggests that this approach can be adapted to directly quantify the translation of the intrahepatic parasite. The OPP labeling technology is particularly flexible, as it does not require any genetic modifications to label the nascent proteome and should thus be directly adaptable to a wide variety of organisms, including other *Plasmodium* species and stages. Supporting this, *P. falciparum* ABS translation can also be visualized in infected erythrocytes using a highly similar protocol (Fig. S2). OPP labeling of nascent polypeptides requires active protein synthesis and should be responsive to chemical inhibition of translation prior to OPP labeling. Treatment of infected HepG2 cells with pan-eukaryotic or *Plasmodium*-specific translation inhibitors recapitulated known inhibitor specificity.

Acute treatment with cycloheximide, which blocks translation elongation via binding the ribosomal E-site (23) and is active against both human and *Plasmodium* translation (24, 25), results in loss of the OPP-A555 signal, indicating a dramatic drop in protein synthesis of both HepG2 and parasite (Fig. 1B). DDD107498 is a *Plasmodium*-specific translation inhibitor thought to target eEF2 (11), and treatment results in loss of OPP-A555 signal only in the parasite, with host HepG2 and parasite nascent proteome (OPP-A55 signal) clearly separable with confocal microscopy (Fig. 1B; Fig. S3). Taken together, our data suggest that OPP labeling of the nascent proteome will allow separate quantification of *Plasmodium* LS translation and that of the host HepG2 cells, thus opening up the study of chemical inhibitors of translation beyond the *Plasmodium* ABS.

### Quantification of the *P. berghei* and HepG2 nascent proteomes

To move from visualization to quantification of the nascent proteome, we utilized automated confocal feedback microscopy (ACFM) (26) to generate unbiased confocal image sets of single *P. berghei* LS parasites and the HepG2 cells immediately surrounding them (referred to as in-image HepG2). Image sets consisted of three separately acquired channels with anti-HSP70 marking the exoerythrocytic parasite forms (EEFs), Hoechst-labeled DNA, and OPP-A555 labeling the nascent proteome in both HepG2 and EEF. We established a CellProfiler (27) pipeline for batch image processing, in which an EEF object segmented in the anti-HSP70 image was then used to mask the other two images for further segmentation and feature extraction (Fig. S4), including fluorescence intensity metrics describing the magnitude of parasite translation via the OPP-A555 signal. To quantify the in-image HepG2 nascent proteome regardless of OPP-A555 signal intensity, we used segmented HepG2 nuclei from the Hoechst image to define the pixels within which to quantify OPP-A55, as this measurement is tightly correlated (*R* = 0.94) with the full cellular HepG2 OPP-A555 signal in control images (Fig. S5). With an image segmentation and feature extraction pipeline in place, we returned to OPP-A555 labeling controls to establish the detectable range of signal specific to the nascent proteome in both *P. berghei* EEFs and in-image HepG2 cells. Infected cells that received no OPP but were subjected to a click labeling reaction with A555 had a larger signal than those that were OPP labeled without fluorophore conjugation. Both were extremely small, though, relative to the specific signal from parasite and HepG2 nascent proteomes, allowing us to specifically quantify translation over a range of ≥3 log units (Fig. S6).

### Assessing LS translation inhibition by compounds with diverse mechanisms of action

**Fig 2:**
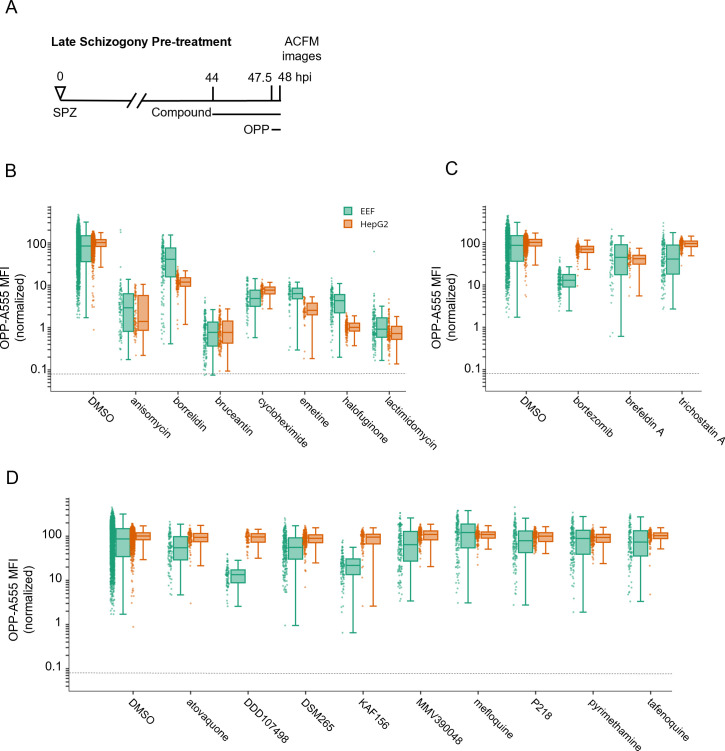
Testing select bioactive compounds for inhibition of *Plasmodium* LS translation. Quantification of *P. berghei* and HepG2 protein synthesis after 3.5 hours pre-treatment with diverse, active compounds, as schematized in A. See Table S-1 for compound details. (**B–D**) Boxplots quantifying translation inhibition via OPP-A555 mean fluorescence intensity [OPP-A555 mean fluorescence intensity (MFI)] from all single parasite ACFM images acquired for *n* ≥ 3 independent experiments, with each dot corresponding to a single EEF (green) or associated in-image HepG2 cells (orange). Specific signal cutoff (see Fig. S2 and S3) is indicated by the dashed line. Compounds tested are known pan-eukaryotic translation inhibitors (B), pan-eukaryotic inhibitors of cellular processes other than translation (C), and antimalarial compounds (D). Compounds were tested at 10 µM except for cycloheximide (10 µg/mL), tafenoquine (1.25 µM), mefloquine (2.5 µM), trichostatin A (5 µM), and brefeldin A (5 µg/mL).

Having established a robust assay to quantify the *P. berghei* LS nascent proteome, we next tested a select set of compounds, including 9 antimalarials and 10 pan-eukaryotic bioactive compounds, for their ability to inhibit *P. berghei* LS translation. The pan-eukaryotic actives include seven compounds that are known translation inhibitors and three compounds with different mechanisms of action (Table S1). While all the pan-eukaryotic compounds have demonstrated antiplasmodial activity against *P. falciparum* ABSs in either growth or re-invasion assays where compounds are present throughout 48+ hours (28–34), comparable data for their LS activity cannot be generated due to the confounding effects that such compounds have on HepG2 cell viability (Fig. S7). To avoid confounding effects of long-term treatment, we first tested compounds for ability to inhibit *P*. berghei LS translation after an acute pre-treatment of 3.5 hours followed by 30 minutes of OPP labeling in the continued presence of test compound (Fig. 2A). Each of the 19 compounds were tested at micromolar concentrations expected to be saturating (Table S1), but which did not induce visible HepG2 toxicity, such as cell detachment or rounding up during 4 hours. Six of the seven pan-eukaryotic translation inhibitors tested inhibited *P. berghei* LS translation by ≥90%, and as expected, the same six translation inhibitors reduced HepG2 translation by ≥90% (Fig. 2B). In contrast, treatment with the threonyl-tRNA synthetase (ThrS) inhibitor borrelidin (35, 36) caused only a 53% mean reduction *P. berghei* LS translation and an 88% mean reduction in HepG2 translation (Fig. 2B). Differences in efficacy of human and *Plasmodium* translation inhibition were also detected for several other compounds. Halofuginone, an inhibitor of *P. falciparum* prolyl-tRNA synthetase (37), and emetine, which inhibits *P. falciparum* elongation (38), both displayed greater efficacy against HepG2 than *P. berghei*, while cycloheximide was slightly more effective against the parasite (Fig. 2B). Bruceantin, an A-site binding elongation inhibitor which does not efficiently inhibit translating polysomes (39, 40), and the E-site-binding elongation inhibitors anisomycin and lactimidomycin (23, 24) caused similar levels of translation inhibition between *P. berghei* and HepG2 cells (Fig. 2B).

To probe assay specificity, we tested three compounds known to be highly active against HepG2 and *Plasmodium* with cellular modes of action other than translation inhibition, which led to complete HepG2 toxicity within 48 hours (Fig. S7). Surprisingly, the 26S proteasome inhibitor bortezomib (41) caused an 86% reduction in *P. berghei* LS translation but had little effect on HepG2 translation (Fig. 2C). Trichostatin A, a histone deacetylase inhibitor (42), and Brefeldin A (BFA), which blocks the secretory pathway in *P. berghei* LS and *P. falciparum* ABS (43, 44), inhibited LS translation by 44% and 46%, respectively (Fig. 2C; Table S1). The third group of test compounds consisted of known antimalarials, with all but mefloquine known to be active against *Plasmodium* LSs (45). None of these antimalarials affected HepG2 translation following acute pre-treatment (Fig. 2D), but two substantially reduced LS translation. DDD107498 (cabamiquine, M5717), thought to act via eEF2 inhibition and a known translation inhibitor in *P. falciparum* ABS (11), inhibited LS translation by 86% (Fig. 2D). KAF156 (ganaplacide), thought to affect the secretory pathway at the level of the ER or Golgi (46, 47), was unexpectedly active in the assay, reducing mean *P. berghei* translation by 76% (Fig. 2D). Three antimalarial compounds caused only slight decreases in *P. berghei* translation following a 3.5-hour acute pre-treatment, including atovaquone, which targets the bc1 complex (48), DSM265, a *Plasmodium* DHODH inhibitor (49), and MMV390048, which targets *Plasmodium* PI4K (50). The remaining antimalarial compounds had little or no effect on parasite translation and included *Plasmodium* DHFR inhibitors pyrimethamine (51) and P218 (52), the 8-aminoquinalone tafenoquine, which lacks a clear mechanism (53), and mefloquine, thought to target *Plasmodium* blood stage feeding but also proposed to inhibit the ribosome (54, 55). All compounds inhibiting *P. berghei* or HepG2 translation by at least 50% were considered active and progressed to concentration-response analysis.

**Fig 3:**
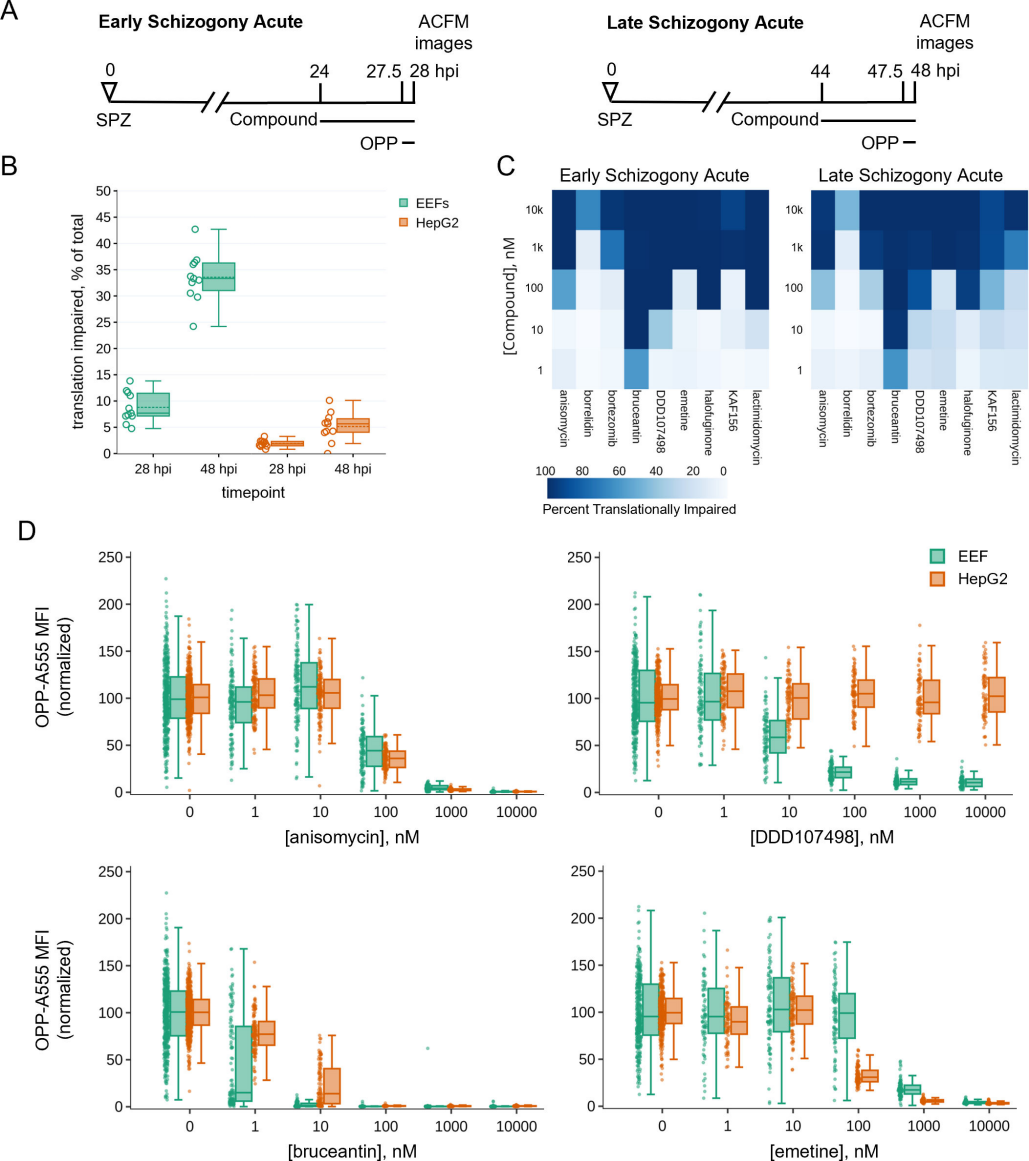
Assessing heterogeneity and potency of translation inhibition during early and late schizogony. (**A**) Experimental schematics. (**B**) DMSO treated control EEFs and corresponding in-image HepG2 cells were classed as translationally impaired (individual parasite OPP-A555 MFI ≤ 50% of experiment OPP-A555 mean) or unimpaired during early and late schizogony; data show the mean of 11 matched independent experiments with circles representing individual experiment values. (**C and D**) Determining potency of translation inhibition in *P. berghei* and in-image HepG2 cells after acute pre-treatment with the inhibitors identified in Fig. 2; *n* ≥ 3 independent experiments. (C) Percentage of single EEFs categorized as translationally impaired for inhibitors across concentrations in early and late schizogony. (**D**) Concentration-response data in single parasites and in-image HepG2 cells for select translation inhibitors; all data normalized to mean of in-plate DMSO controls, set to 100.

We initially chose to run the acute pre-treatment assay during late schizogony due to the advantages of imaging larger parasites but found that a substantial number of control parasites had translational outputs resembling those pre-treated with translation inhibitors (Fig. 2). Given that all *Plasmodium* LSs do not successfully complete development *in vitro* (56, 57), we performed the concentration-response experiments during both early and late schizogony in parallel (Fig. 3A) to additionally probe for developmental differences in parasite translation. Using 11 paired data sets, raw mean translation intensity in 28 vs 48 hpi parasites was significantly different while that of in-image HepG2 was not [*P* = 0.00019 (LS), 0.0995 (HepG2); paired *t*-test]. We defined individual parasites as “translationally impaired” if the OPP-A555 mean fluorescence intensity (MFI) was ≤50% of the mean OPP-A555 of all in-plate DMSO controls and similarly classified the in-image HepG2. Using this definition of translational impairment, there is a substantial increase in translationally impaired control LSs at 48 hpi (33.3%) vs 28 hpi (7.7%), while a more modest shift was seen in the HepG2 (Fig. 3B). On average, parasite size was highly similar between translationally impaired and unimpaired parasites at 28 hpi but markedly different at 48 hpi (Fig. S8, *P* < 0.005), suggesting that translational impairment in 48hpi control parasites is indicative of earlier developmental failure or growth inhibition.

Despite the marked difference in translational heterogeneity between the parasite populations at 28 and 48 hpi, both efficacy and potency of the 10 compounds active against LS protein synthesis were quite similar in early vs late schizogony and reproducible across independent experiments (Fig. 3C; Fig. S9 and S10). Anisomycin, which blocks elongation by occupying the A-site and preventing peptide bond formation (23, 58), has very similar potency against human and *P. berghei* translation, while DDD107498 is completely parasite-specific, as expected (Fig. 3D). Modest selectivity toward *P. berghei* is seen for bruceantin, the most potent inhibitor tested, while emetine has greater potency against HepG2 protein synthesis (Fig. 3D; Fig. S9 and S10). For all pan-eukaryotic translation inhibitors tested except borrelidin, which only achieved 64% inhibition at the maximum concentration tested against early LSs, translation inhibition efficacy was similar between HepG2 and *Plasmodium* (Fig. 3D; Fig. S9 and S10). Lactimidomycin lost potency against both *Plasmodium* and HepG2 translation during late schizogony (Fig. 3C; Fig. S9 and S10); this likely reflects compound instability (see Methods). Anisomycin, bruceantin, cycloheximide, halofuginone, and lactimidomycin all inhibited protein synthesis in early LS schizonts by >95% (Table S2) after 3.5 hours of treatment, despite their varied modes/mechanisms of action. DDD107498 reached only 90.5% inhibition with the same treatment duration at the highest dose tested, despite having clearly achieved a saturating response (Fig. 3D; Table S2). Concentration-dependent inhibition of LS translation was seen for both KAF156 and bortezomib, which reached 77% and 86% inhibition, respectively (Table S2).

### Differentiating known direct translation inhibitors from compounds indirectly inhibiting LS translation

The acute pre-treatment assay was designed to maximize signal from translation inhibitors while avoiding confounding effects from HepG2 toxicity often seen with long treatment windows. However, this means that the assay will identify both direct translation inhibitors, and those that inhibit translation indirectly during the period of acute pre-treatment, such as compounds that induce cellular stress, eventually leading to a signaling-based shutdown of protein synthesis, e.g. through phosphorylation of eIF2α (59), or those that are capable of rapidly killing *P. berghei* LSs within the 3.5-hour window when the compound is present before OPP labeling. To test whether we could distinguish the known direct translation inhibitors from the other active compounds we identified, we implemented a competition OPP assay (co-OPP), where OPP and the compound of interest are added to *P. berghei*-infected HepG2 monolayers concomitantly for 30 minutes. Since puromycin analogs like OPP truncate a nascent polypeptide chain at the position they are incorporated, the co-OPP assay effectively means there is a possibility for direct competition between the test compound and OPP to shut down translation of each nascent polypeptide at each codon (17, 20–22). The known direct translation inhibitors should reduce OPP-A555 labeling of the nascent proteome competitively within the brief labeling period, with those mechanistically expected to compete with OPP at each translating codon likely to display similar potency and efficacy as in the acute pre-treatment assay, while compounds such as bruceantin and halofuginone, that directly inhibit protein synthesis but not at each translating codon, would be predicted to be at slight disadvantages.

**Fig 4:**
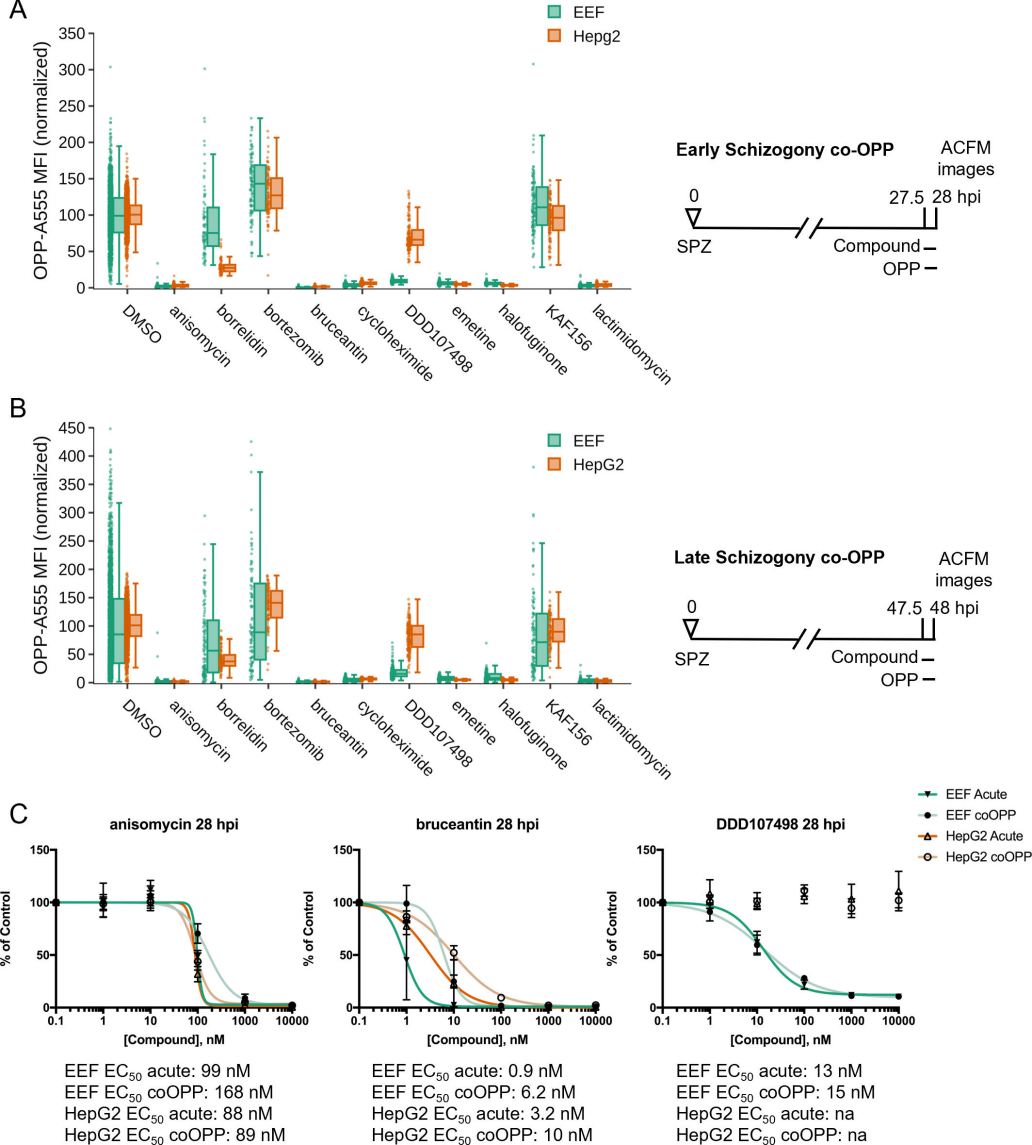
Identification of direct vs indirect translation inhibitors. Quantification of protein synthesis in *P. berghei*-infected HepG2 cells in which active compounds at maximal concentrations were added together with OPP in early (**A**) and late (**B**) schizogony as described in figure schematics. Compound concentrations tested are the same as in Fig. 2. Each data point represents the normalized OPP-A555 mean fluorescence intensity (OPP-A555 MFI) of a single EEF or the corresponding HepG2 cells as labeled. (**C**) Comparing co-OPP and acute pre-treatment (from Fig. S3-1) concentration-response curves. All data shown was collected in *n* ≥ 3 independent experiments.

The co-OPP assay was first run at top concentration (see Table S1) during both early and late *P. berghei* schizogony. Strikingly, both KAF156 and bortezomib, the two unexpected actives in acute pre-treatment mode, were not competitive inhibitors of OPP labeling at either timepoint and are thus indirect translation inhibitors, though the nature of their indirect translation inhibition is unknown (Fig. 4A and B). Here, bortezomib treatment increased translational intensity in HepG2 cells at both timepoints and in early *P. berghei* schizonts (Fig. 4A and B; Table S2). Anisomycin, bruceantin, cycloheximide, emetine, halofuginone, and lactimidomycin were all direct inhibitors of both *P. berghei* and HepG2 protein synthesis, while DDD107498 was a direct inhibitor of parasite translation only (Fig. 4A and B). Anisomycin and DDD107498, thought to act against the elongation step of protein synthesis, and bruceantin, which inhibits the peptidyl transferase center but has low affinity for polysomes, were selected for 5pt. 10-fold serial dilution dose response to test whether any difference in potency could be detected in co-OPP vs acute pre-treatment assays in early *P. berghei* LS schizonts. Bruceantin showed a clear reduction in parasite translation inhibition potency in the competition assay, with a ~sixfold shift in EC_50_, while DDD107498 and anisomycin did not (Fig. 4C; Table S2). The success of the competition assay in identifying all known direct inhibitors of HepG2 or *Plasmodium* ABS translation suggests that the co-OPP assay can be useful to identify antimalarial compounds of unknown mechanism that likely function as direct translation inhibitors.

### Investigation of the mechanism of indirect translation inhibition by bortezomib and KAF156

**Fig 5:**
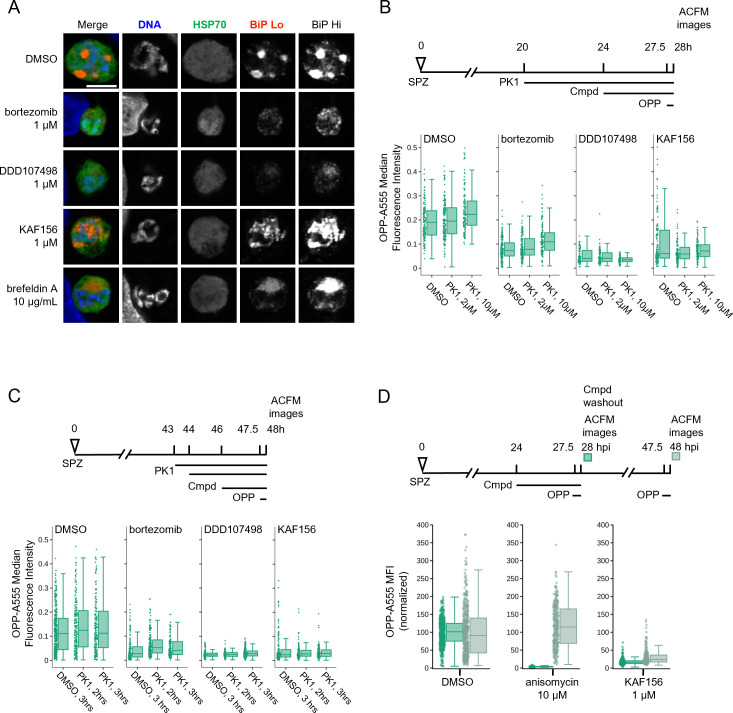
Investigating potential mechanisms behind indirect translation inhibition. (**A**) Representative, single confocal images of *P. berghei* LS ER morphology after 4-hour compound treatment in early schizogony, at 28 hpi. Single channel images, all acquired with identical settings, are shown in grayscale, with merges pseudocolored as labeled; HSP70 marks the parasite and BiP specifically labels the parasite ER. Two images of BiP immunofluorescence were acquired with different gains (BiP Lo and BiP Hi) to visualize ER morphology across the range of BiP intensity observed. All images are shown at the same scale; scale bar = 5 µm. (**B–D**) Quantification of protein synthesis in single EEFs following treatments detailed in associated schematics. In (B and C) (bortezomib) = 1 µM, (KAF156) = 0.5 µM, and (DDD107498) = 0.1 µM were used to achieve similar levels of submaximal translational inhibition in the parasites. *n* ≥ 3 independent experiments. PK1 as labeled in B, and 20 µM in C. Data in (D) was normalized to the mean of the DMSO control parasites for each timepoint.

Our finding that bortezomib and KAF156 similarly caused indirect translation inhibition in *P. berghei* LSs was unexpected, as they have distinct modes of action. They may, however, converge phenotypically downstream of endoplasmic reticulum (ER) stress, as bortezomib-driven accumulation of misfolded or damaged proteins in the ER causes an unfolded protein response (UPR) that is partially conserved in *Plasmodium* (60), while multiple lines of evidence indicate that KAF156 affects the parasite ER (46, 61). To investigate whether ER stress might be driving the indirect translation inhibition caused by KAF156 and bortezomib, we first investigated the phenotypic impact of both compounds on *P. berghei* LS ER structure using BiP, an HSP70 localized to the ER lumen (62), as a marker in immunofluorescence analysis (IFA). The LS schizont ER is a single, continuous structure composed of ER centers (tight accumulations of tubules) interconnected by a network of thin tubules (63) (Fig. 5A; DMSO). A 4-hour BFA treatment causes these centers to collapse into a single structure, while the immunofluorescence intensity of anti-BiP labeling is similar to the control; DDD107498 treatment led to a similar collapse of ER centers, together with a substantial reduction in BiP IFA signal intensity localized to a single dim ER center (Fig. 5A). Bortezomib and KAF156 both altered the ER morphology profoundly, with the ER appearing to have fragmented or vesiculated throughout the EEF (Fig. 5A). Strikingly, bortezomib also caused a marked reduction in BiP signal intensity, like DDD107948, while KAF156 does not (Fig. 5A). These findings support the hypothesis that KAF156 and bortezomib could both induce ER stress leading to subsequent translational arrest in the *P. berghei* LS.

The *Plasmodium* response to ER stress appears to lack the transcriptional regulatory arm of the eukaryotic UPR (63, 64), but that which attenuates translation via eIF2α phosphorylation is present and active. Three eIF2α kinases exist in *Plasmodium* (65), with PK4 (PBANKA_1126900, PF3D7_0628200) mediating phosphorylation of eIF2α when ER stress is induced by DTT or artemisinin in *P. berghei* and *P. falciparum* ABSs (66–68). *Plasmodium* PK4 appears orthologous to the human PERK kinase, and *P. falciparum* and *P. berghei* PK4 activity can be inhibited by the human PERK inhibitor GSK2606414 (PK1) (66, 67, 69). To test whether indirect translation inhibition caused by bortezomib and KAF156 was mediated by the eIF2α kinase PK4, we tested if PK1 pre-treatment could prevent translation inhibition by these compounds. DDD107498 was used as a control since it inhibits *P. berghei* LS translation directly (Fig. 4), and PK4 inhibition should thus have no effect on its activity. We first tested 4 hours PK1 pre-treatment at 0 (DMSO control), 2, or 10 µM from 20 to 24 hpi, followed by the addition of KAF156 (0.5 µM), bortezomib (1 µM), DDD107498 (0.1 µM), or DMSO from 24 to 28 hpi, with OPP added in the final 30 minutes. These concentrations of KAF156, bortezomib, and DDD107498 were chosen to induce sub-maximal translation inhibition and showed clear, but incomplete reduction in the OPP-A555 median fluorescence intensity in single parasites (Fig. 5B). However, pre-treatment with PK1 did not prevent subsequent inhibition of EEF translation by bortezomib, KAF156, or DDD107498 (Fig. 5B). We also tested a shortened 20 µM PK1 pre-treatment and shortened KAF156 and bortezomib treatments, as prolonged PK1 treatment at this concentration leads to HepG2 cytotoxicity (not shown). Control experiments demonstrate that 2 hours treatments with bortezomib or KAF156 are sufficient to induce translational arrest, but once again, PK1 was not able to prevent translation inhibition by either compound (Fig. 5C). In both PK4 inhibition protocols, in-image HepG2 translation was also quantified. Bortezomib treatment alone led to a reduction in HepG2 translation as has been previously demonstrated, and shown to be mediated by human PERK (70); PERK inhibition by PK1 pre-treatment markedly increased HepG2 translation after addition of bortezomib (Fig. S11). These results demonstrate that the indirect translation inhibition induced by KAF156 and bortezomib is not mediated by *Plasmodium* PK4. Another hypothesis for this indirect translation inhibition is that it reflects a rapid parasite death process. If so, the translation inhibition should not be reversible. We tested this directly by comparing the reversibility of the translation inhibition induced by 4 hours KAF156 and anisomycin treatments in early schizogony. Anisomycin-induced translation inhibition is reversible in human cells (58), and the ~95% inhibition of *P. berghei* LS translation was completely reverted 20 hours after compound washout (Fig. 5D). KAF156 treatment induced weaker translation inhibition (~85%) compared to anisomycin but showed very little recovery of translation 20 hours after washout. The irreversibility of the translation inhibition after washout suggests that KAF156 treatment causes rapid parasite death.

### Testing uncharacterized *P. berghei* LS active compounds for the ability to inhibit protein synthesis

**Fig 6:**
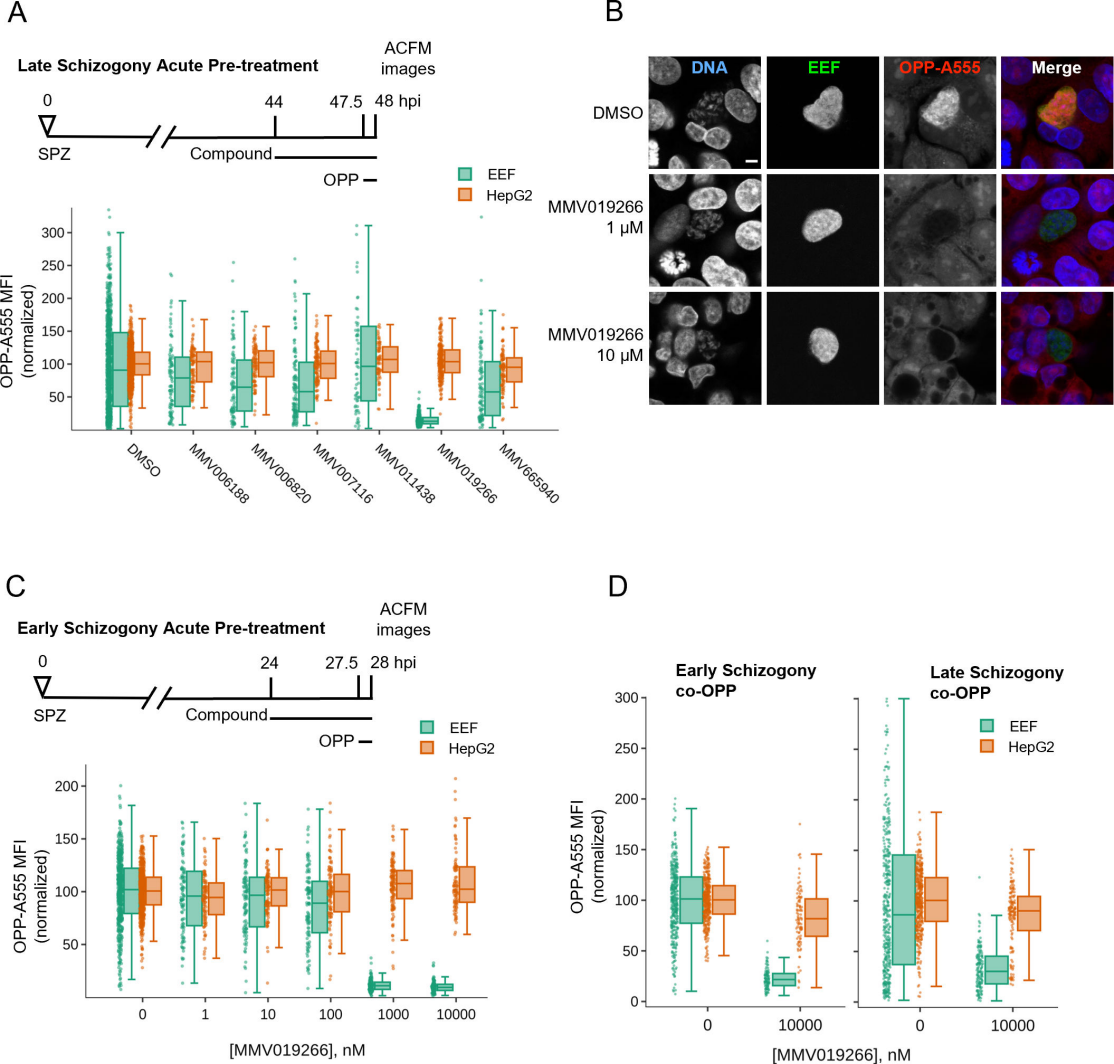
Characterization of MMV019266 inhibition of *P. berghei* LS translation. (**A**) Select LS actives from the Malaria Box were tested at 10 µM for ability to inhibit *P. berghei* LS translation following acute pre-treatment in late schizogony. (**B**) Representative single confocal images of OPP-A55 labeling after 4 hours acute pre-treatments with MMV019266 vs control in late schizogony; merges are pseudocolored as indicated with parasite (EEF) immunolabeled with α-HSP70, and DNA stained with Hoechst. All images are shown at the same scale; scale bar = 5 µm. (**C and D**) Quantification of protein synthesis in *P. berghei-*infected HepG2 cells; compound treatments as described. Each data point represents OPP-A555 MFI normalized to in-plate controls; *n* ≥ 3 independent experiments.

Finally, to investigate the utility of this assay for identifying novel *Plasmodium* protein synthesis inhibitors, we tested six compounds from the MMV Malaria Box that are active against *P. berghei* LSs and phenotypically similar to DDD107498 in 48 hours luciferase assays (71). Acute pre-treatment with MMV019266 reduced EEF translation by 87% (Fig. 6A). The remaining compounds were much less active, with MMV665940, MMV007116, and MMV006820 causing roughly 30% reduction in PbLS translation, MMV006188 causing a 19% reduction, and MMV011438 having no effect (Fig. 6A). MMV019266 similarly inhibited LS translation at both 1 and 10 µM during early and late schizogony (Fig. 6B and C; Fig. S12; Table S2). MMV019266 had EC_50_ values of 373 and 289 nM at 28 and 48 hpi, respectively, in the acute pre-treatment assay (Fig. S12; Table S2). MMV019266 was also capable of inhibiting *P. falciparum* ABS translation in intact schizonts, with a degree of inhibition similar to that seen with 10 µM DDD107498, but greater than 20 nM DDD107498 and less than 100 nM bruceantin (Fig. S13). The co-OPP assay demonstrated that MMV019266 is likely a direct protein synthesis inhibitor, causing 77% and 72% reductions in PbLS translational intensity during early and late schizogony, respectively (Fig. 6D; Table S2). Identification of MMV019266 as a translation inhibitor in both blood stage and LS parasites highlights the utility of the *P. berghei* LS OPP assay to antimalarial drug discovery.

## DISCUSSION

Our results demonstrate the feasibility and utility of single-cell image-based quantification of protein synthesis in an intracellular parasite that resides in a translationally active host cell, and open up the study of *Plasmodium* LS translation for drug discovery applications and in the native developmental context. To date, studies of the mode of action and target of antimalarial compounds largely rely on studies in *P. falciparum* ABSs, with the assumption that it will be the same in other stages and species in which a compound has antiplasmodial activity. Molecular targets of antimalarials compounds have been identified and validated in *P. falciparum* ABS through *in vitro* evolution of drug resistance, cellular thermal shift assays, chemoproteomics, metabolic profiling, and a variety of reverse genetics approaches to produce modified parasite lines (50, 72–74). Re-use of these same evolved or genetically modified parasite lines, metabolic profiling and assays quantifying intracellular ionic concentrations all support further understanding of antimalarial compound modes of action in ABS (16, 75, 76). Protein synthesis in ABS has long been quantified by feeding with radiolabeled amino acids, and more recently, *P. falciparum* ABS lysate assays detecting the translation of a single model transcript encoding a luciferase enzyme have been used to screen several small compound libraries and characterize the activity of known pan-eukaryotic translation inhibitors (14, 15, 77).

An overarching difficulty in the quantification of conserved biochemical or cellular processes like protein synthesis in *Plasmodium* LSs is the dominant contribution of hepatocytes to any signal from an infected monolayer. In ABS, this problem is easily overcome, as saponin lysis of infected red blood cell (RBC) cultures has long been recognized to allow parasite purification (78), and mature human erythrocytes lack most core cellular processes, e.g., protein synthesis, allowing *Plasmodium* translation to be quantified directly in bulk ABS cultures or lysates. The inability to physically isolate LS parasites or isolate the parasite signal from that of the hepatocytes prevents the use of these approaches in *Plasmodium* LSs currently. Here, we overcome this limitation by using computational separation of the combined fluorescent signal of the nascent proteome of infected HepG2 monolayers into separate *P. berghei* and hepatoma cells signals in ACFM-acquired image sets. The specificity of this approach is clear from *Plasmodium*-specific inhibition of translation by DDD107498, and our ability to detect differential inhibition of *H. sapiens* vs. *P. berghei* translation with pan-eukaryotic inhibitors like emetine and bruceantin means that both host and parasite nascent proteomes can be quantified in parallel, allowing determination of a compound’s LS translation inhibition efficacy and selectivity in a single well. Similar quantitative bioimaging strategies may prove useful for drug discovery efforts with other eukaryotic parasites residing in translational active host cells.

One attractive feature of targeting *Plasmodium* translation is that such inhibitors would be predicted to have multistage activity, as has been demonstrated for DDD107498 and a variety of tRNA synthetase inhibitors (11, 79). Though our data show that DDD107498 LS translation inhibition potency is 13–15 nM, less than its ~1–2 nM LS antiplasmodial potency in standard 48-hour LS biomass assays, it is clearly a concentration-dependent translation inhibitor. It is striking, though, that at 1 nM, we detect no clear translation inhibition at all, and the slope is very shallow, with saturating effects only seen at 1,000 nM, and the percent max translation inhibition is less than for other parasite-active compounds. These effects seem unlikely to be time dependent, as we show nearly identical translational responses to DDD107498 in acute pre-treatment and co-OPP assays. Incomplete translation inhibition with saturating doses of DDD107498 was also seen in *P. falciparum* ABS (11), and it will be a future challenge to determine how much translation inhibition is required for DDD107498 antiplasmodial activity in both liver and blood stage parasites, and indeed, that of other antimalarial compounds which also inhibit translation, but with different molecular mechanisms. Consistent with the hypothesis that translation inhibitors should be multistage actives, we show concentration-dependent LS inhibition for the elongation inhibitors anisomycin, lactimidomycin, emetine, and cycloheximide, the initiation inhibitor bruceantin, and the tRNA synthetase inhibitor halofuginone. Only borrelidin, a known inhibitor of ThrS in both prokaryotes and eukaryotes (35), failed to show concentration-dependent translation inhibition activity against *P. berghei* LSs and was only partially effective in our acute pre-treatment assay at the 10 µM, a concentration that is overwhelmingly cytotoxic to hepatocytes in a 48-hour treatment. This partial translation inhibition activity at micromolar concentrations is at odds with the low nanomolar antiplasmodial potency of borrelidin against ABS, with reported IC_50s_ ranging from 0.07 to 1.9 nM (29, 36, 80–82). We tested borreldin in a 48-hour LS live luciferase assay, but all concentrations that reduced parasite biomass also showed effects on the HepG2 monolayer (data not shown), so it is unclear if borrelidin has any direct antiplasmodial activity against the *P. berghei* LS. Species-specific differences in activity should not be the cause, as borrelidin was active against ABS of both human and murine *Plasmodium* spp., and was an effective antimalarial in murine infection models (29, 36, 80, 81). Furthermore, this disconnect is not easily explained by stage-specific differences in the target enzyme expression or activity, as *Plasmodium* parasites encode only a single copy of ThrS (83), which is likely required for protein synthesis in both the cytoplasm and apicoplast (59, 84). Enzymatic evidence clearly shows that borrelidin is active against recombinant PfThrS *in vitro* (36), but the cellular evidence in support of borrelidin targeting *Plasmodium* ThrS was a modest shift in the *P. falciparum* ABS growth inhibition EC_50_ when an excess of exogenous free L-threonine in growth media (80). Evolved *in vitro* resistance to borrelidin has not been reported to date. Given that compound efficacy against a molecular target *in vitro* is not always a reliable indicator of *in vivo* antimalarial mechanism, as with triclosan (85), it will be important to clarify that ThrS is indeed the relevant antimalarial target of borrelidin, and if so, understand why it is not effective against *P. berghei* LS translation.

The image-based OPP assay appears to have some advantages relative to the lysate assay, PfIVT (14, 15, 77), in testing antiplasmodial compounds of unknown mechanism for translation inhibition activity. Given the liquid handling requirements for the OPP assay, it is ideally suited for use with adherent cells, and thus LS parasites, and while our current 96wp format is sufficient for testing of compounds of interest as we demonstrate here, we are miniaturizing the assay to 384 wp format for medium throughput use. Image-based assays have the advantage of the inherent ability to investigate the ground-truth of translation inhibition metrics obtained via segmentation and feature extraction, as metadata links the original, unaltered image set to extracted features (86), while a lysate-based assay lacks inherent ground truth, and may require a secondary counterscreen to triage compounds against the translated reporter enzyme, as for PfIVT (77). Translation is a complex process, requiring spatial coordination of hundreds of gene products (59, 87) to produce new proteins from thousands of mRNAs, and the image-based OPP assay captures changes in output of the entire, native nascent proteome. A lysate-based assay using translation of a single exogenous mRNA as a readout reduces this complexity substantially and may fail to identify compounds that are active translation inhibitors *in cellulo*. Perhaps this occurred with MMV019266, which was not identified as an active compound in the PfIVT screen of the Malaria Box (14). We tested six compounds identified from the MMV Malaria Box as LS active with a 48-hour biomass assay phenotype indicative of early LS arrest (71), the same as for DDD107498, which we could source commercially. While five were inactive, the thienopyrimidine MMV019266 was identified as a likely direct translation inhibitor in *P. berghei* LS, and we demonstrated that it also inhibits *P. falciparum* translation in blood stage schizonts. MMV019266 is known to have antiplasmodial activity against a variety of species and life cycle stages, including *Plasmodium vivax* schizonts and *P. falciparum* gametocytes (71, 88–92), and was predicted to target hemoglobin catabolism based on metabolic fingerprinting (93). During the preparation of this manuscript, three related thienopyrimidines were reported to target the *P. falciparum* cytoplasmic isoleucyl tRNA synthetase (PfcIRS) based on mutations evolved *in vitro* in resistant lines and confirmed in conditional PfcIRS knockdowns and gene-edited parasite lines (94). These results highlight the value of the *P. berghei* LS OPP assay for the identification of multistage *Plasmodium* translation inhibitors.

While our focus here has been using the quantitative image-based OPP assay to identify novel *Plasmodium* translation inhibitors and validate this mode of action in the LS for known inhibitors of *P. falciparum* blood stage translation, the flexibility of the assay and power of single-cell data suggest it may prove quite useful in key applications beyond this. While our quantitative work utilized LS schizonts, we demonstrated specific nascent proteome signal in sporozoites through to monolayer merozoites and intriguing changes in the subcellular localization of the nascent proteome seem to occur during *P. berghei* LS development. The difference in the fraction of translationally impaired parasites in 28 vs 48 hpi LS schizonts suggests that translational intensity may vary during LS development in populations, as it clearly does in individuals at both timepoints. We identify Kaf156 and bortezomib as indirect translation inhibitors in the acute pre-treatment assay and show that their activity is not under the control of the eIF2α kinase PK4, though signaling-based inhibition of eIF2α via phosphorylation by a different kinase or perhaps eEF2 phosphorylation, which can be mediated by CDPK3 in *Toxoplasma gondii* (95), remain potential mechanisms. However, our data showing an overwhelming lack of reversibility of translation inhibition after KAF156 washout lead us to favor the hypothesis that these compounds are causing rapid killing of *P. berghei* LS schizonts and indicate the potential of using translational output as a biomarker for LS parasite viability, something that remains lacking in the LS toolkit (96). The labeling protocol adapts easily to *P. falciparum* blood stages, as we demonstrate, though throughput is limited by the non-adherent erythrocytes, which also complicates high-content quantitative imaging. While the throughput problem will be challenging to solve for medium to high throughput drug discovery, flow cytometry may be better suited to quantification of translation inhibition in based on fluorescent labeling of the *P. falciparum* nascent proteome, as has recently been done to characterize novel tyrosine-RNA synthetase inhibitors (82). As OPP labeling of the nascent proteome requires no transgenic technology, it should be readily adaptable to critical drug discovery challenges such as testing target engagement and potency of translation inhibitors with diverse molecular mechanisms of action against field isolates of *P. falciparum* and *P. vivax*. Our quantitative bioimaging workflow should be repurposable for interrogation of translation inhibitors in *P. falciparum* and *P. vivax* LSs and capable of integration into existing image-based-screening platforms (97) and may have particular value in examining the role translation plays in the formation of dormant hypnozoites and their reactivation.

The OPP-based cellular bioimaging assay we present here also has limitations for the identification of novel compounds as direct *Plasmodium* translation inhibitors. The assay, whether run with an acute pre-treatment or in competition mode, only provides information about protein synthesis at the cellular level and, as such, can never provide definitive proof that a compound directly inhibits translation, which would require functional studies at the molecular level. The co-OPP assay was able to distinguish between known direct translation inhibitors and all other bioactive compounds, even those that were quite active against *P. berghei* LS translation after acute pre-treatment, but we cannot exclude that indirect inhibitors of translation, regardless of mechanism, with very rapid inhibition kinetics might show similar activity in acute pre-treatment and co-OPP assays. Compounds that are unable to cross the lipid bilayers of the host hepatocyte plasma membrane, the parasitophorous vacuole membrane, and the parasite plasma membrane would not be active in this assay but could be recognized as active in lysate-based assays. While we expect that true inhibitors of the translation initiation step would be detected as active in the assay, none were tested here. Finally, the assay would not detect compounds that affect the fidelity of translation, such as the majority of inhibitors affecting the termination step, which lead to mis-coding and stop codon read through (98), unless they also reduced the amount of translation occurring in the parasite.

## MATERIALS AND METHODS

### HepG2 culture and *P. berghei* sporozoite isolation and infection

HepG2 human hepatoma cells were cultured in Dulbeco’s Modified Eagle Medium (DMEM; Gibco 10313–021) supplemented with 10% (vol/vol) fetal bovine serum, 1% (vol/vol) GlutaMAX (Gibco 35050–061), 1% (vol/vol) Penicillin-Streptomycin (Gibco 15140–122), and maintained at 37°C, 5% CO_2_. *P. berghei* sporozoites, expressing firefly luciferase-GFP fusion protein under the control of the exoerythrocytic form 1 a (EEF1a) promoter (99), were isolated from the salivary glands of infected *Anopheles stephensii* mosquitos (NYU and UGA insectaries). Sporozoites were counted and diluted into infection DMEM (iDMEM) – cDMEM further supplemented with 1% (vol/vol) Penicillin-Streptomycin-Neomycin (Gibco 15640–055), 0.835 µg/mL Amphotericin B (Gibco 15290–018), 500 µg/mL kanamycin (Corning 30–006-CF), and 50 µg/mL gentamycin (Gibco 15750–060), added to HepG2 monolayers, centrifuged at 3,000 rpm for 5 minutes, and incubated in cell culture conditions for 2 hours before phosphate buffered saline (PBS) washing and iDMEM replenishment for infections proceeding on glass coverslips. For infections in 96 well plates (Greiner 655098), infected HepG2 monolayers were detached at 2 hpi using TrypLE Express (Gibco 12605–028), washed, counted, and re-seeded into 96 well plates.

### Compound handling and treatment

Compound stocks prepared from powder were solubilized in DMSO (Sigma-Aldrich D2650), aliquoted, and stored at −20°C. For acute pre-treatments, infected cells were treated for 3.5 hours prior to 30 minutes OPP labeling in the continued presence of the compound. For co-OPP assays, compound and OPP were applied simultaneously for 30 minutes. Concentration-response experiments were performed with five points in 10-fold serial dilutions, and equimolar DMSO concentrations [0.001% (vol/vol)] were maintained across all treatments and controls. Compounds prepared in iDMEM were stored at 4°C and used within 24 hours, e.g., a single dilution series was prepared and used for both the 24 and 44 hpi additions. For the 48 hpi recovery timepoint in Fig. 5D, the acute treatments were removed at 28 hpi, and coverslips were washed 3× with iDMEM and returned to the incubator for subsequent processing.

### OPP labeling and fluorophore addition

A 20 mM stock of OPP (Invitrogen C10459) in DMSO was diluted to label cells with 20 µM OPP for 30 minutes at 37°C, according to the manufacturer’s recommended protocol, before 15 minutes fixation with paraformaldehyde (PFA) (Alfa Aesar 30525–89-4) diluted to 4% in PBS. Copper-(I)-catalyzed cycloaddition of Alexafluor555 picolyl azide to OPP-labeled polypeptides was performed using Invitrogen Click-iT Plus AF555 (Invitrogen C10642) according to the manufacturer’s recommendations, with a 1:4 Cu_2_SO_4_ to copper protectant ratio. 27 µL of reaction mix was added to 96-wp wells, with 25 µL used for each glass coverslip, inverted on parafilm.

### Immunofluorescence

EEFs were immunolabeled using anti-PbHSP70 (2E6 mouse mAb) (100) (1:200), followed by donkey anti-mouse Alexafluor488 (Invitrogen A21202). In Fig. 1A, goat anti-UIS4 (Sicgen AB0042-500) (1:1,000) was used to mark the newly invaded sporozoites, followed by donkey anti-goat 488 (Invitrogen A32814). To visualize the parasite ER in Fig. 5A, rabbit polyclonal anti-BiP (1:600, GenScript) serum, raised against the C terminal polypeptide CGANTPPPGDEDVDS from PBANKA_081890, was used with donkey anti-rabbit Alexafluorr555 (Invitrogen A31572) as the secondary. DNA was stained with Hoescht 33342 (Thermo Scientific 62249) (1:1000). Antibodies were prepared in 2% BSA in PBS, with secondary antibodies used at a 1:500 dilution.

### *P. falciparum* culture

*P. falciparum* 3D7 parasites were cultured as previously described (101). In short, parasites were cultured in human AB^+^ erythrocytes (Interstate Blood Bank, Memphis, TN, USA) at 3%–10% parasitemia in a complete culture medium (5% hematocrit). Complete culture medium consisted of RPMI 1,640 medium (Gibco #32404014) supplemented with gentamicin (45 µg/mL final concentration; Gibco #15710064), HEPES (40 mM; Fisher #BP3101), NaHCO_3_ (1.9 mg/mL; Sigma #SX03201), NaOH (2.7 mM; Fisher #SS266-1), hypoxanthine (17 µg/mL; Alfa Aesar #A11481-06), L-glutamine (2.1 mM; Corning #25005 Cl), D-glucose (2.1 mg/mL; Fisher #D16-1), and 10% (vol/vol) human AB^+^ serum (Valley Biomedical #HP1022). Parasites were cultured at 37°C in an atmosphere of 5% O_2_, 5% CO_2_, and 90% N_2_.

### *P. falciparum* blood stage immunofluorescence and OPP-A555 labeling

*P. falciparum*-infected erythrocytes (iRBCs) in mixed culture were labeled using 20 µM OPP (Invitrogen C10459) at 37°C for 30 minutes, pelleted, and washed with PBS before being resuspended in 1 mL of 4% PFA (Electron Microscopy Sciences 30525–89-4) +0.0075% glutaraldehyde (Sigma G6257) in PBS for 30 minutes at room temperature (RT). Fixed iRBCs were pelleted and washed twice with PBS prior to permeabilization in 0.1% Triton X-100 (9002–93-1) in PBS for 10 minutes. Permeabilized iRBCs were washed twice in PBS, click-labeled as described for infected HepG2 monolayers, then pelleted, washed once with PBS, and Hoechst-labeled for 30 minutes at RT. iRBCs were then pelleted, washed, and resuspended in PBS before imaging.

### HepG2 viability assay

Non-infected HepG2 cells were treated with 10-point, threefold, serial dilutions with maximal concentrations of 10 µM, except for cycloheximide (10 µg/mL), GSK260414 (50 µM), and emetine (25 µM). At 46 hours post-treatment, AlamarBlue cell viability reagent (Invitrogen A50100) was applied at a 1X final concentration and incubated for 1 hour prior to measuring fluorescence at 590 nm using a microplate reader (CLARIOstar, BMG LABTECH).

### Image acquisition

Images were acquired on a Leica SP8 confocal microscope using an HC PL APO 63×/1.40 oil objective for glass coverslips and an HC PL APO 63×/1.40 water objective for 96-well μclear plates. Images in Fig. 1A, 5C and 7B; Fig. S1 to S3 were acquired manually and processed using ImageJ (102). All other images, and all used for quantitative analysis, were acquired using ACFM (26). Briefly, MatrixScreener is used to define a patterned matrix for the acquisition of non-overlapping, low-resolution images of the *P. berghei-*infected HepG2 monolayer. After each image is acquired, online image segmentation and ID of parasites, defined by PbHSP70 signal, is performed utilizing custom modules (https://github.com/VolkerH/MatrixScreenerCellprofiler/wiki) integrated into a CellProfiler version 2.0.11710 pipeline (27). The x-y coordinates of each parasite found are then used by MatrixScreener to sequentially image each individual parasite in high resolution, with an automated z-stack maximizing PbHSP70 intensity to identify the z coordinate, followed by sequential acquisition of Hoechst, PbHSP70, and OPP-A55 images. This process iterates until all parasites in the predefined matrix of the infected monolayer have been imaged.

### Image segmentation, feature extraction, and data cleaning

Batch image segmentation and feature extraction were performed in Cell Profiler (v2.1.1 rev6c2d896) (27); see Fig. S4 for the workflow. Briefly, EEF objects were identified using a global Otsu segmentation of the PbHSP70 image. The EEF object was shrunk by two pixels to ensure the exclusion of HepG2-associated signal and used to mask the OPP-A555 image to quantify *P. berghei* translation via OPP-A555 fluorescence intensity features. Conversely, the EEF object was expanded by two pixels and used as an inverse mask for the Hoechst image to segment HepG2 nuclei. All in-image HepG2 nuclei were unified into a single object, then its OPP-A555 fluorescence intensity features were used to quantify HepG2 translation. All features extracted were then analyzed using KNIME (103). ACFM image sets were computationally cleaned of image that did not contain a single true EEF in a HepG2 monolayer by removing data from those in which: more than one EEF object was identified, the EEF object identified did not contain a DNA signal, or no HepG2 nuclei were identified. EEF object form factor was used to identify rare instances of segmentation failure in which two parasites were segmented as a single EEF object; images set corresponding to form factor outliers (>1.5× IQR) were visually inspected and removed if they did not contain a single, true EEF. Finally, focus score features for both the PbHSP70 and DNA images were used to exclude any image set where the focus score <1.5 IQR. Data cleaning was carried out per experiment and resulted, on average, in the removal of 1.45% of the total acquired data.

### Concentration response curve fitting and statistics

Concentration-response analysis was performed using four parameter non-linear regression curve fitting in GraphPad Prism (Version 7.0d), with the top of the curve fixed at 100, and −10 < hill slope <0. When maximal effect was reached with ≥2 concentrations tested, the bottom of the curve was fit open; if no such plateau was achieved, the curve was fit with maximal effect constrained to 0. EC_50_ and 95% CI were determined for each compound from ≥3 independent experiments. All other data and statistical analyses were performed in KNIME.

## Data Availability

The image data sets analyzed are available upon request to the corresponding author.

## SUPPLEMENTAL MATERIAL

Supplemental Figures msphere.00544-23-s0001.pdf Figures S1-S6.

Additional Supplemental Figures msphere.00544-23-s0002.pdf Figures S7-S13.

Table S1 msphere.00544-23-s0003.xlsx Compounds.

Table S2 msphere.00544-23-s0004.xlsx Compound potency and efficacy of translation inhibition.

ASM does not own the copyrights to Supplemental Material that may be linked to, or accessed through, an article. The authors have granted ASM a non-exclusive, world-wide license to publish the Supplemental Material files. Please contact the corresponding author directly for reuse.
