# Supplementary material for: Single-cell quantitative bioimaging of P. berghei liver stage translation
Source: mSphere. 2023 Nov 1;8(6):e00544-23. doi: 10.1128/msphere.00544-23 (PMC10732057; doi:10.1128/msphere.00544-23)
Supplement: Supplemental Figures — Figures S1-S6. [file msphere.00544-23-s0001.pdf]

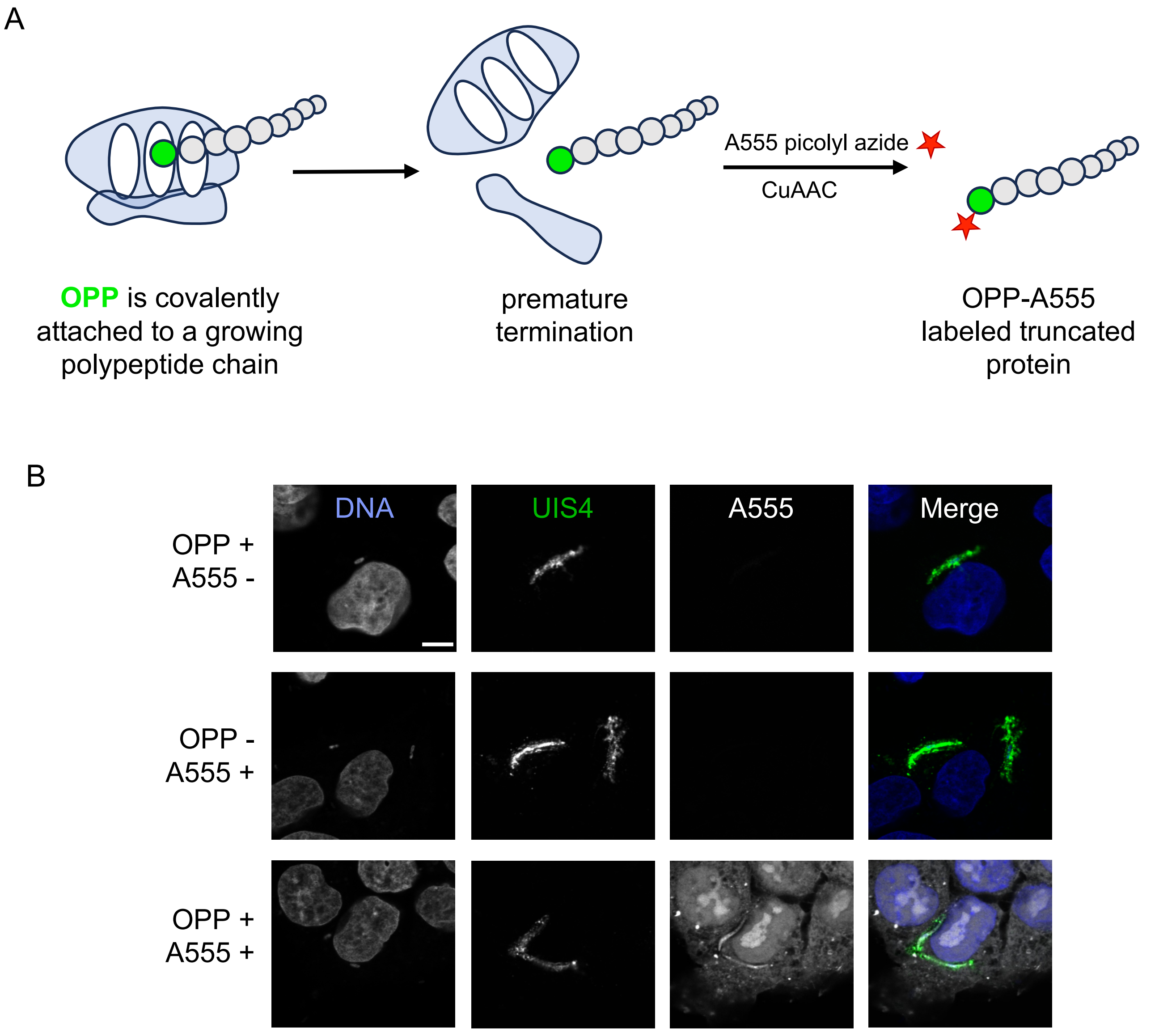

Figure S1. Specificity of the OPP-A555 nascent proteome signal. A) Schematic illustrating OPP incorporation into a nascent polypeptide on a translating ribosome, leading to premature termination of translation and release of the OPP-labeled polypeptide.Post-fixation, OPP-labeled polypeptides are visualized following a copper catalyzed azide-alkyne cycloaddition (CuAAC) of a fluorescent picolyl azide. B) Control experiments were performed to visualize the specificity of OPP-A555 labeling of *P. berghei*-infected HepG2 at 2 hpi. Infected cells were incubated with (OPP+) or without (OPP-) OPP for 30 minutes before fixation. Following fixation, click labeling reactions were performed with (AF555+) and without (AF555-) addition of fluorophore to the labeling reaction mix. Representative, single confocal images were acquired and processed with identical settings. Single channel images are shown in grayscale, merged images are pseudo colored as labeled, with α-UIS4 marking the parasite and Hoechst staining DNA. All images are shown at the same scale; scale bar = 5 μm.

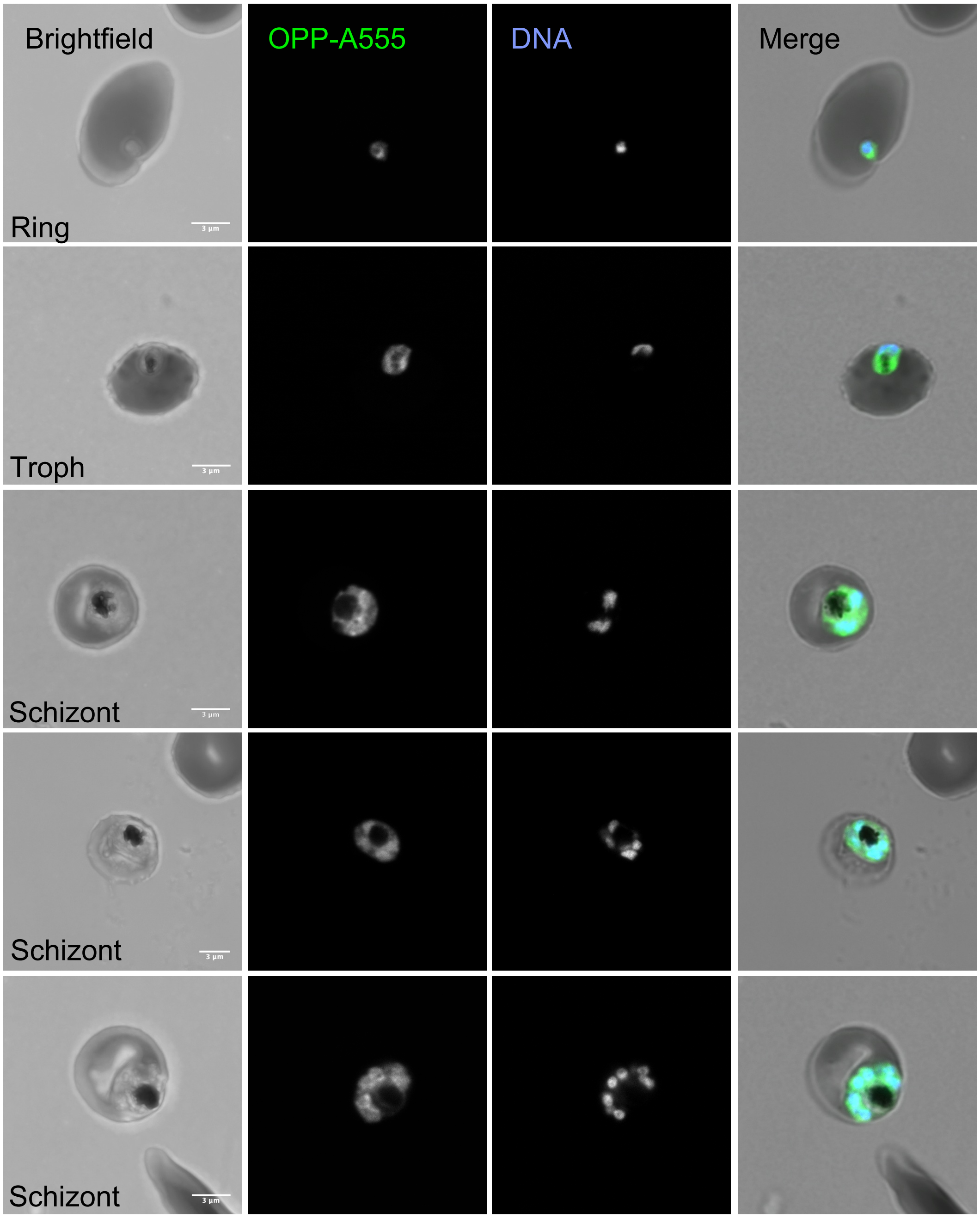

Figure S2. Visualization of the nascent proteome in *Plasmodium falciparum* asexual blood stage parasites. Representative single confocal images (fluorescence and merge) and maximum intensity projections (brightfield) of *Plasmodium falciparum*-infected erythrocytes with OPP-A555 labeling the nascent proteome and Hoechst labeling DNA. Scale bars displayed for each image set = 3 μm.

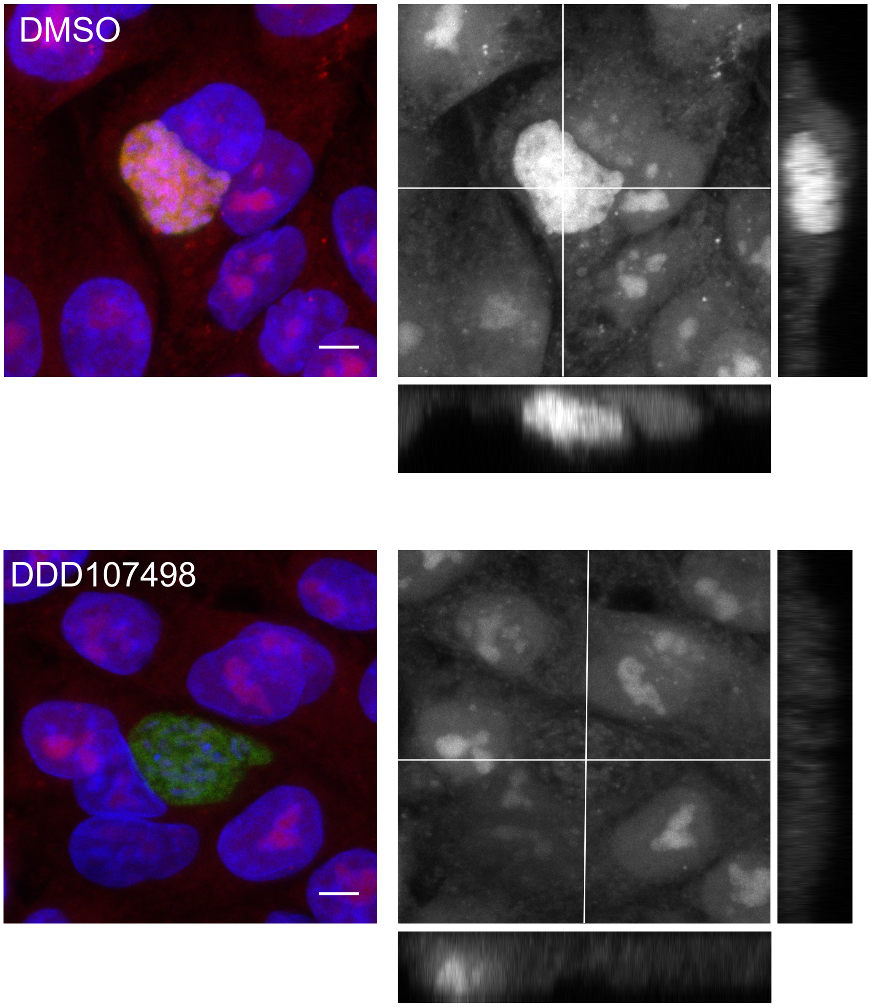

Figure S3. OPP-A555 fluorescence in the host hepatocyte does not preclude quantification of the parasite signal in confocal images. Confocal z-stacks through *P. berghei*-infected HepG2 cells with OPP-A555 labeling the nascent proteome following acute pre-treatment, are shown in maximum intensity projections in grayscale (xy) flanked by xz and yz confocal stacks at the positions indicated by intersecting lines in the xy projection. Image stacks are comprised of 11 (DMSO) and 9 (DDD107498) confocal images acquired with a 1μm z-step. Merged images are pseudo colored with OPP-A555 labeling the nascent proteome in red, Hoechst staining DNA in blue, and α-PbHSP70 immunolabeling the EEF in green. The xy images shown illustrate the the slice that would be algorithmically chosen for ACFM imaging to maximize the intensity of the PbHSP70 signal. Scale bars = 5 μm.

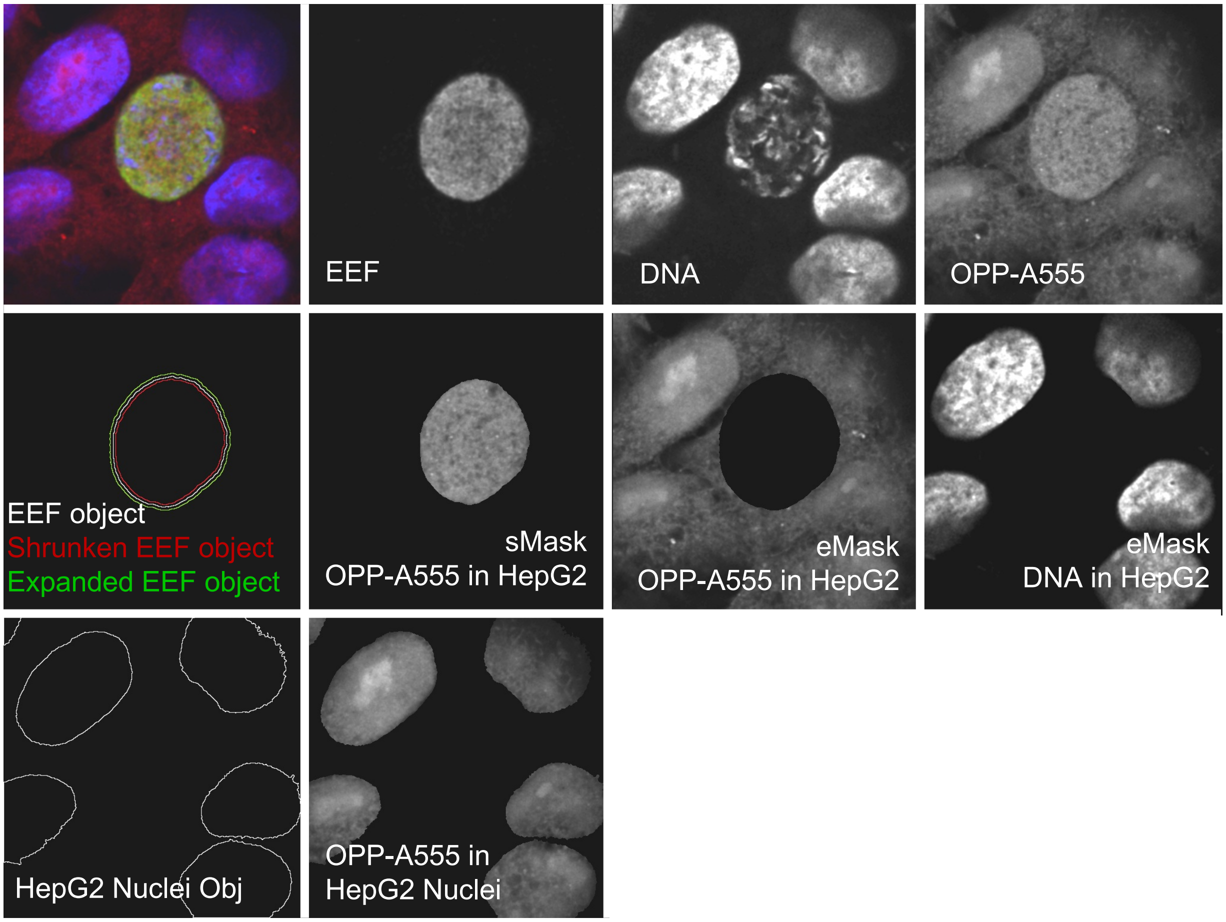

Figure S4. Overview of image segmentation and masking for specific quantification of host and parasite protein synthesis. After ACFM image acquisition, the EEF image (anti-HSP70) was segmented in CellProfiler to identify the set of labeled pixels, defined as the EEF object. The EEF object is then computationally shrunk and expanded by 2 pixels to generate the shrunken EEF and expanded EEF objects respectively, which exclude the host-parasite interface pixels. The shrunken EEF object is used to mask DNA and OPP-A555 (sMask as shown) for further segmentation and/or quantification of parasite features. The expanded EEF object is used as an inverted mask (eMask as shown) to select all pixels outside the parasite, allowing identification of HepG2 nuclei objects, and quantification of features inside the HepG2 cells.

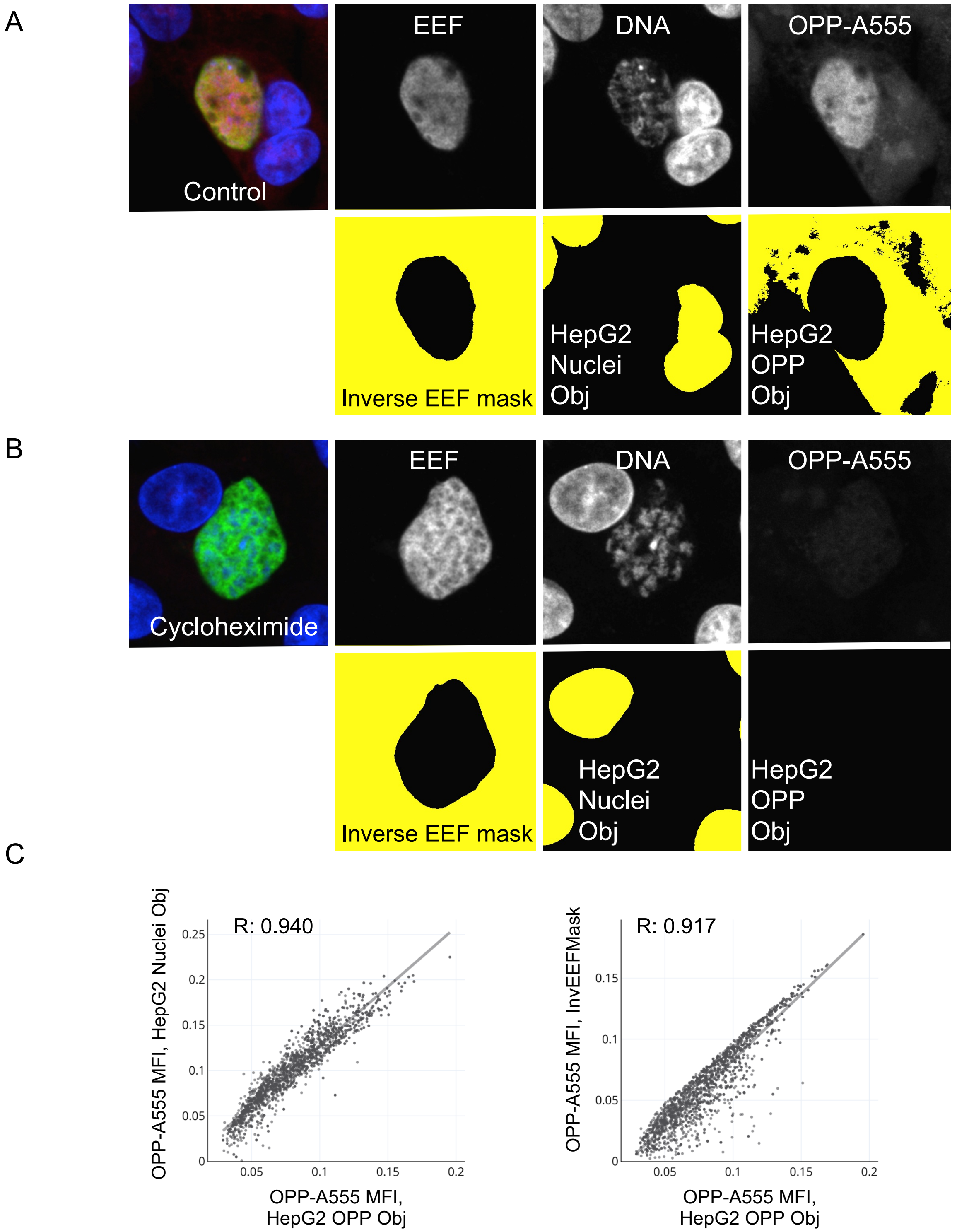

Figure S5. The OPP-A555 signal in HepG2 nuclei is well correlated with whole cell translation intensity. HepG2 protein synthesis was quantified in confocal ACFM images of *P. berghei*-infected HepG2 cells using three different strategies. Pixels marked for quantification are pseudo colored yellow in A-B). While the actual HepG2 OPP-A555 signal can be easily segmented (HepG2 OPP Obj) control cells (A), this method fails for images where HepG2 translation has been blocked by an inhibitor such as cycloheximide (B). Two additional strategies not dependent on segmentation of the OPP-A555 signal also implemented for comparison to the translation intensity of the HepG2 OPP Obj: quantification of OPP-A555 signal in all pixels of the inverse EEF mask (InvEEFMask) and in the segmented HepG2 nuclei (HepG2 Nuclei Obj), as shown in A-B). C) Correlation of OPP-A555 MFI from the HepG2 OPP Obj with that of HepG2 Nuclei Obj and InvEEFMask using a control parasite dataset consisting of 3779 single parasite ACFM images (single dot) from 13 independent infections, all with 44-48 hpi DMSO treatment, OPP labeling, and fixation at 48 hpi. R (correlation) is indicated for each.

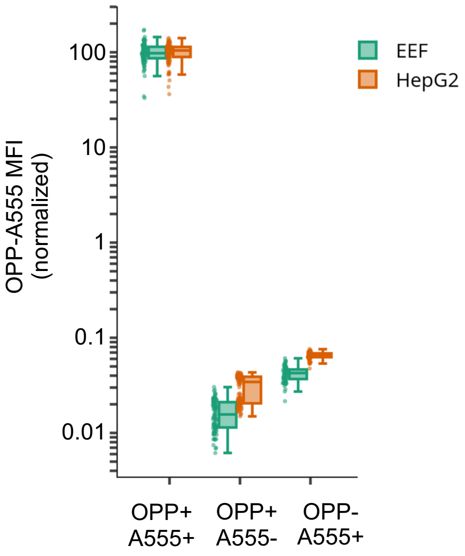

Figure S6. Quantifying signal specificity for OPP-A55 labeling of the nascent proteome. *P. berghei* -infected HepG2 cells were labeled with OPP (OPP+) or DMSO (OPP-) for 30 minutes before fixation at 28 hpi. Following fixation, click reactions were performed with (A555+) and without (A555-) addition of the fluorophore to the labeling reaction mix, followed by immunolabeling of parasites and DNA staining. Images were acquired with ACFM using the OPP+ A555+ to determine acquisition settings used for all conditions, and the mean of this condition was set to 100 and used to normalize all data. Boxplots quantify the amount of signal detected with and without the OPP and fluorophore addition, with each point corresponding to a single parasite, or the associated in-image HepG2 cells. Data is from a single experiment.
