# Supplementary material for: Single-cell quantitative bioimaging of P. berghei liver stage translation
Source: mSphere. 2023 Nov 1;8(6):e00544-23. doi: 10.1128/msphere.00544-23 (PMC10732057; doi:10.1128/msphere.00544-23)
Supplement: Additional Supplemental Figures — Figures S7-S13. [file msphere.00544-23-s0002.pdf]

A

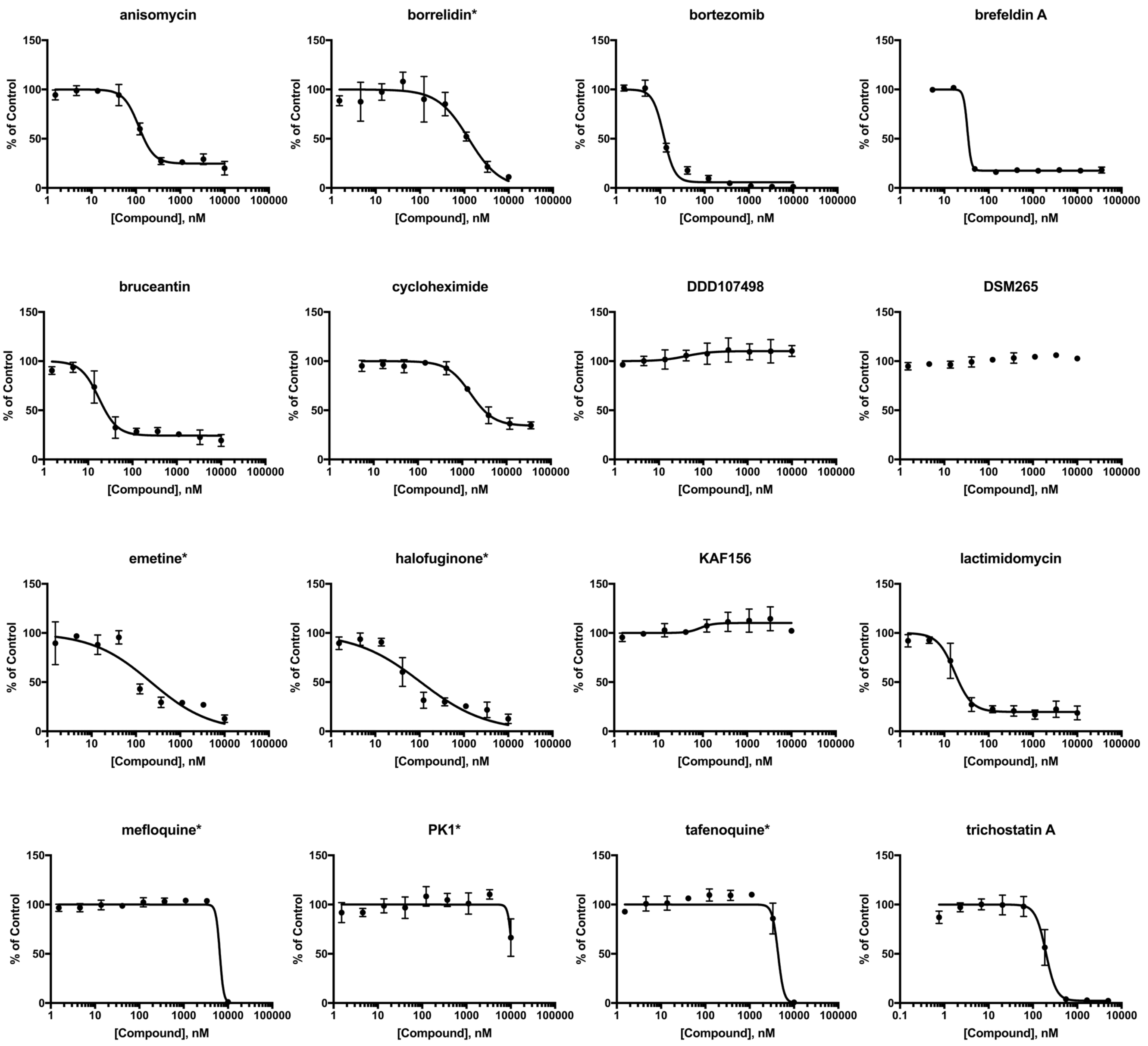

Figure S7. HepG2 cytotoxicity of compounds tested for ability to inhibit liver stage translation. Cellular viability of uninfected HepG2 cells determined by AlamarBlue fluorescence following a 48h compound treatment with 3-fold serial dilution; starting maximal concentration 10μM, except cycloheximide (10μg/mL). Concentration-response curves marked with an asterisk were fit with the bottom of the curve constrained to 0, while all others were fit open as detailed in Materials and Methods. Points represent the mean of 3 independent experiments, and errors bars show standard deviation.

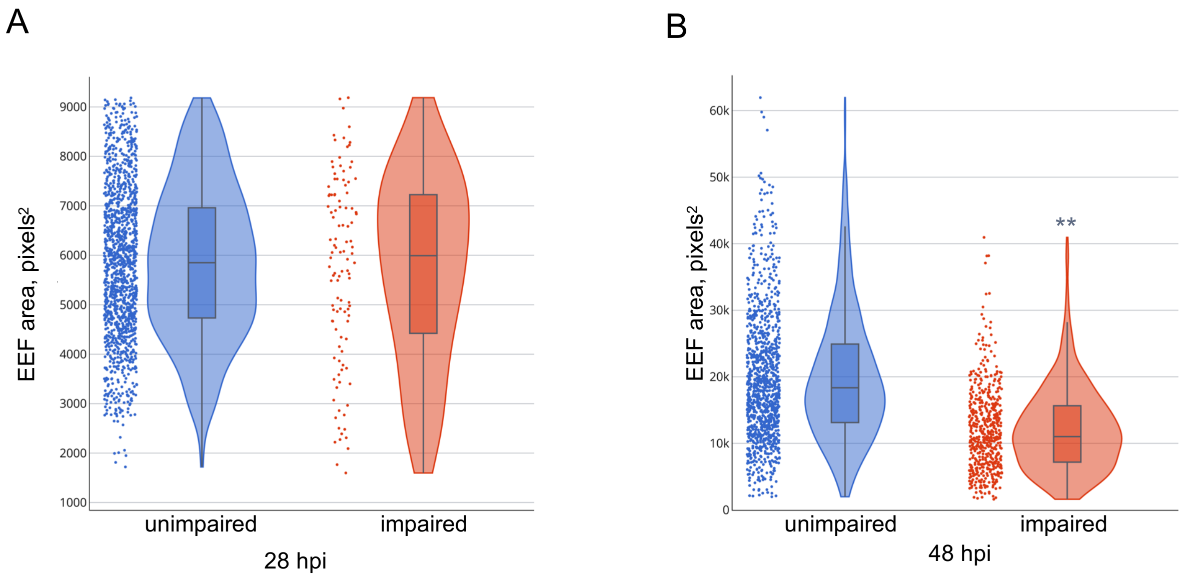

Figure S8. Quantifying the relationship between parasite size and translation intensity in control EEFs. A-B) Area of single, DMSO-treated control parasites, classed as translationally impaired or unimpaired (Fig. 3B dataset, see Fig. 3 legend for classification definition) at 28- and 48 hpi. To ensure that only images containing a single EEF were analyzed at 28 hpi, parasites with an area larger than the 9th decile for the entire dataset in Fig. A (9188 $pixels^2$) were excluded from the analysis. No filtering was applied to the 48 hpi data, as wide variation in parasite size exists at this timepoint. Paired, two-tailed t-tests were run on mean area (unnormalized) of EEFs assigned to either translation class, from a total of 11 matched independent experiments. ** = $p < 0.005$.

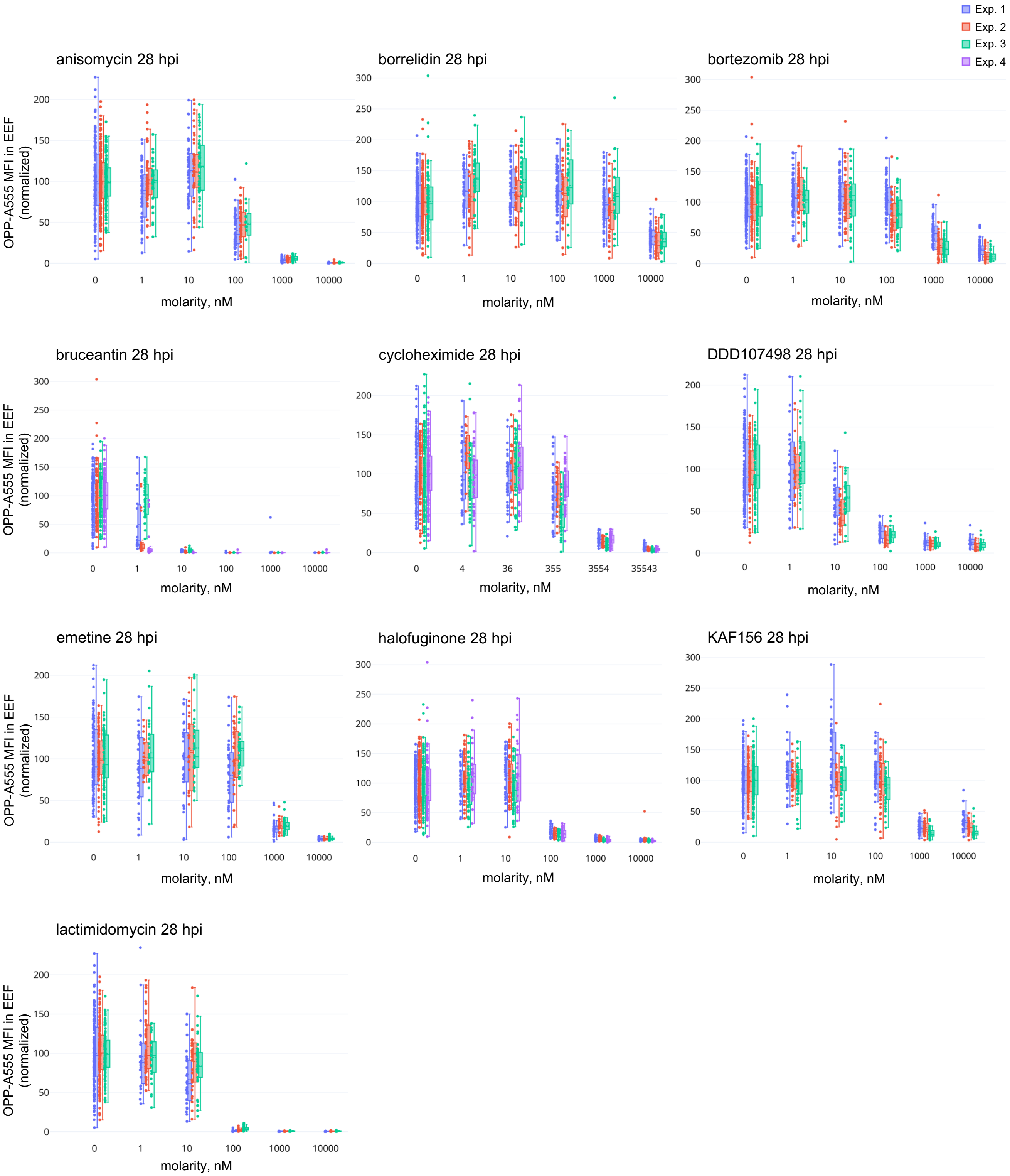

Exp. 1
Exp. 2
Exp. 3
Exp. 4
anisomycin 28 hpi
borrelidin 28 hpi
bortezomib 28 hpi
bruceantin 28 hpi
cycloheximide 28 hpi
DDD107498 28 hpi
emetine 28 hpi
halofuginone 28 hpi
KAF156 28 hpi
lactimidomycin 28 hpi
OPP-A555 MFI in EEF (normalized)
molarity, nM
0 1 10 100 1000 10000
0 4 36 355 3554 35543

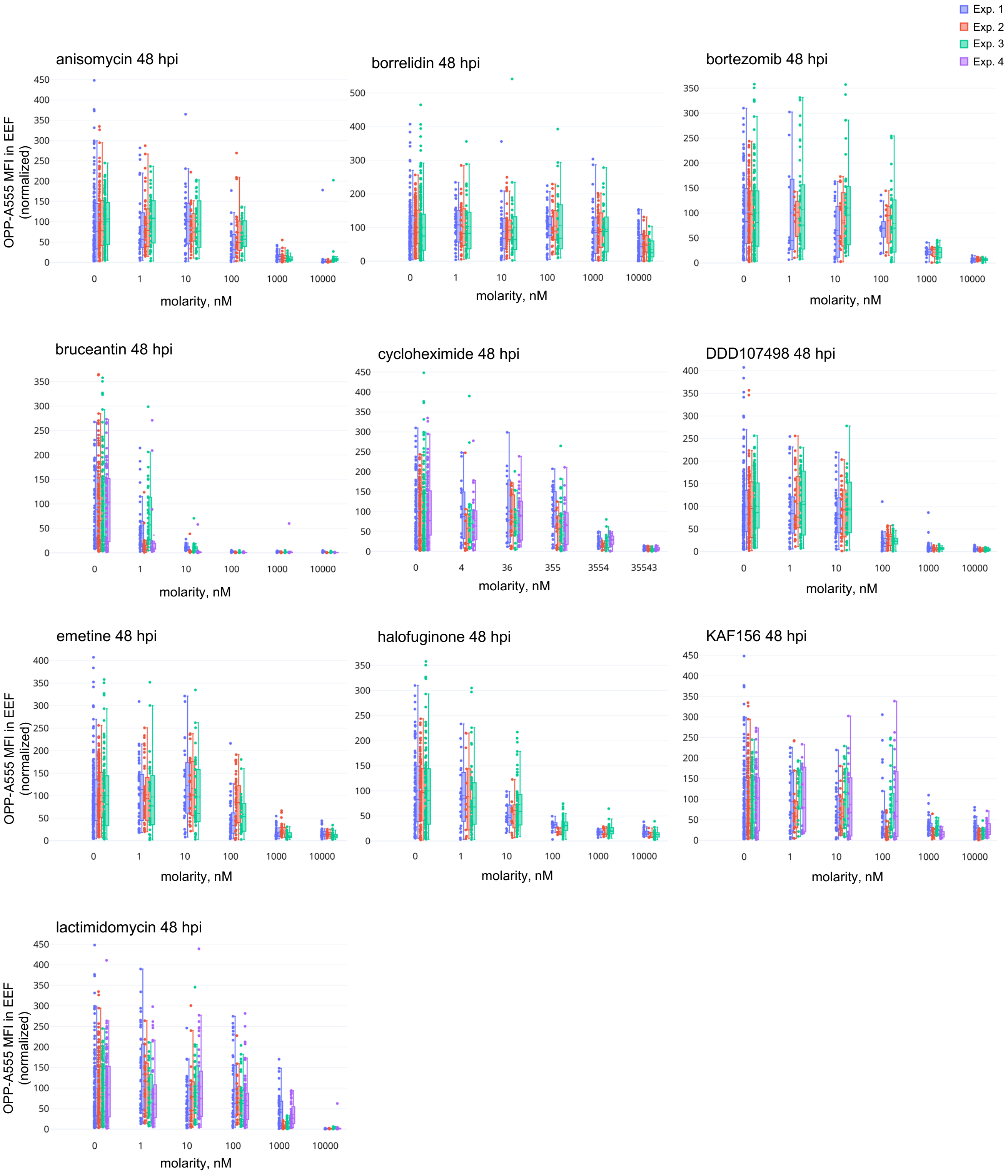

Figure S9. Concentration dependent translation inhibition in EEFs by experiment. Protein synthesis (OPP-A555 MFI) in single EEFs was quantified and normalized to in-plate DMSO controls following acute pre-treatment with a 5-point, 10-fold serial dilution with timepoint and compound treatment as labeled. Same data shown in Fig. 3C; here, data for each independent experiment is (n≥3) shown separately.

A

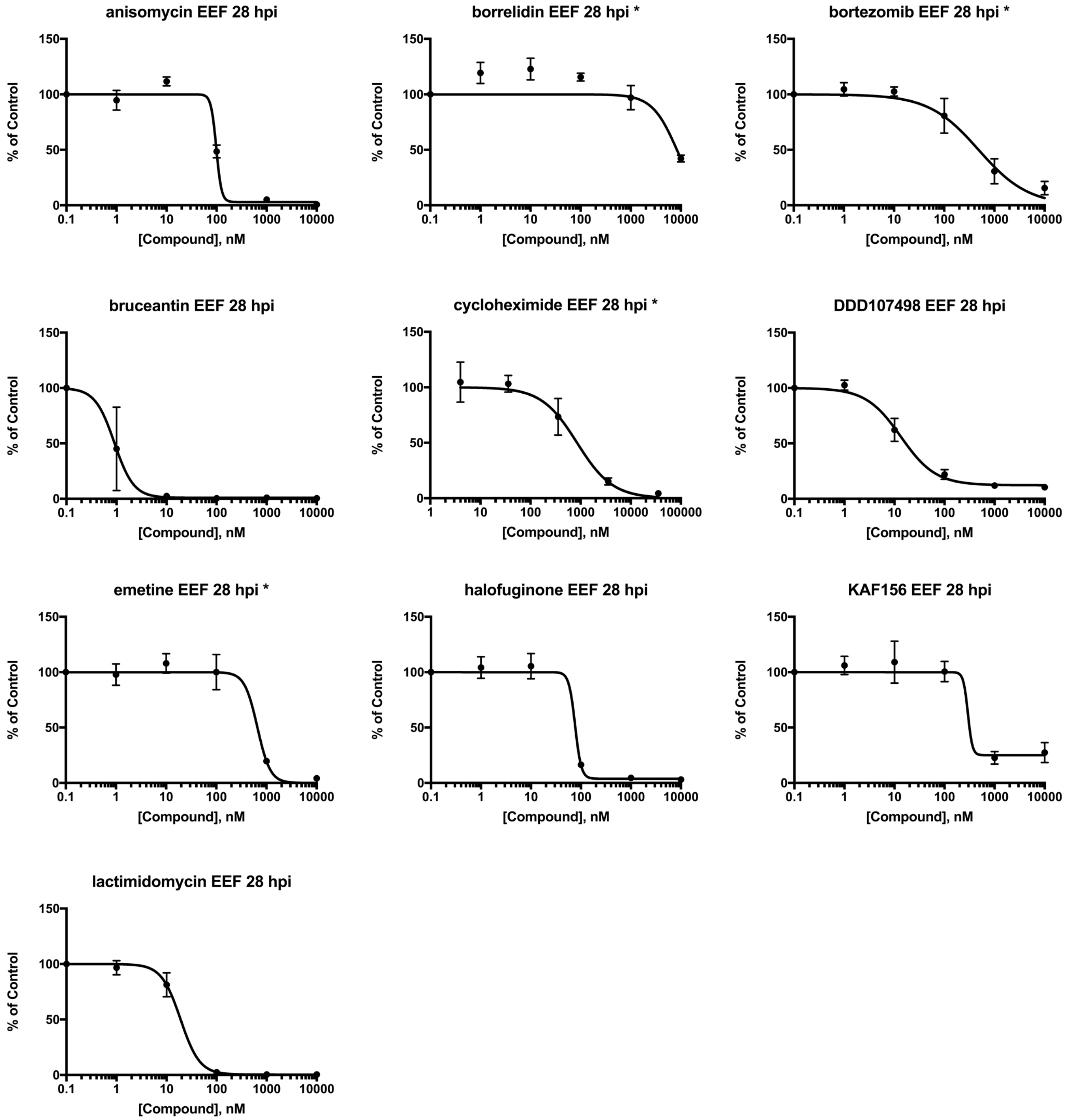

anisomycin EEF 28 hpi
borrelidin EEF 28 hpi *
bortezomib EEF 28 hpi *
bruceantin EEF 28 hpi
cycloheximide EEF 28 hpi *
DDD107498 EEF 28 hpi
emetine EEF 28 hpi *
halofuginone EEF 28 hpi
KAF156 EEF 28 hpi
lactimidomycin EEF 28 hpi
% of Control
150
100
50
0
0.1
1
10
100
1000
10000
100000
[Compound], nM

B

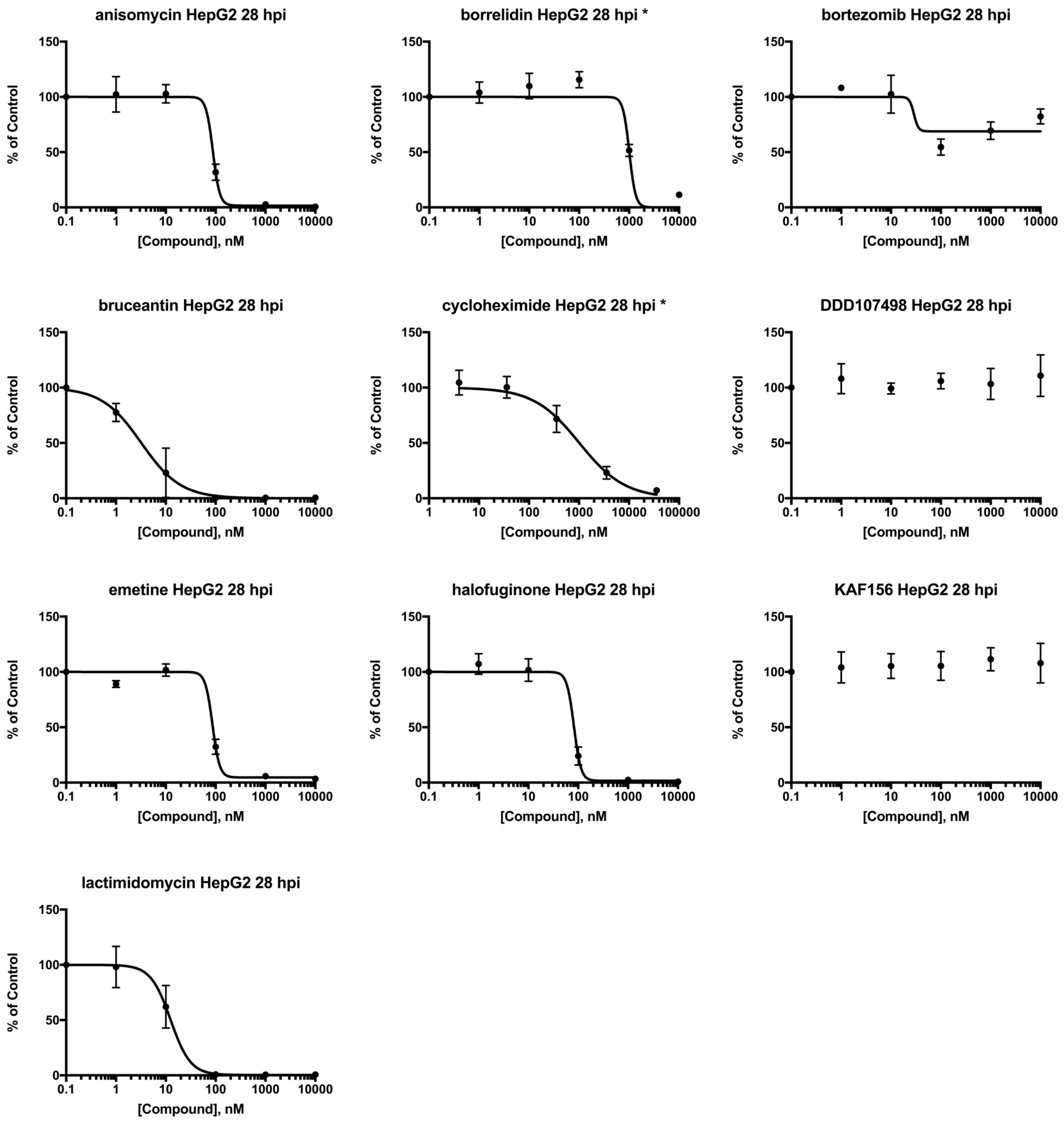
anisomycin HepG2 28 hpi
borrelidin HepG2 28 hpi *
bortezomib HepG2 28 hpi
bruceantin HepG2 28 hpi
cycloheximide HepG2 28 hpi *
DDD107498 HepG2 28 hpi
emetine HepG2 28 hpi
halofuginone HepG2 28 hpi
KAF156 HepG2 28 hpi
lactimidomycin HepG2 28 hpi
% of Control
[Compound], nM

C

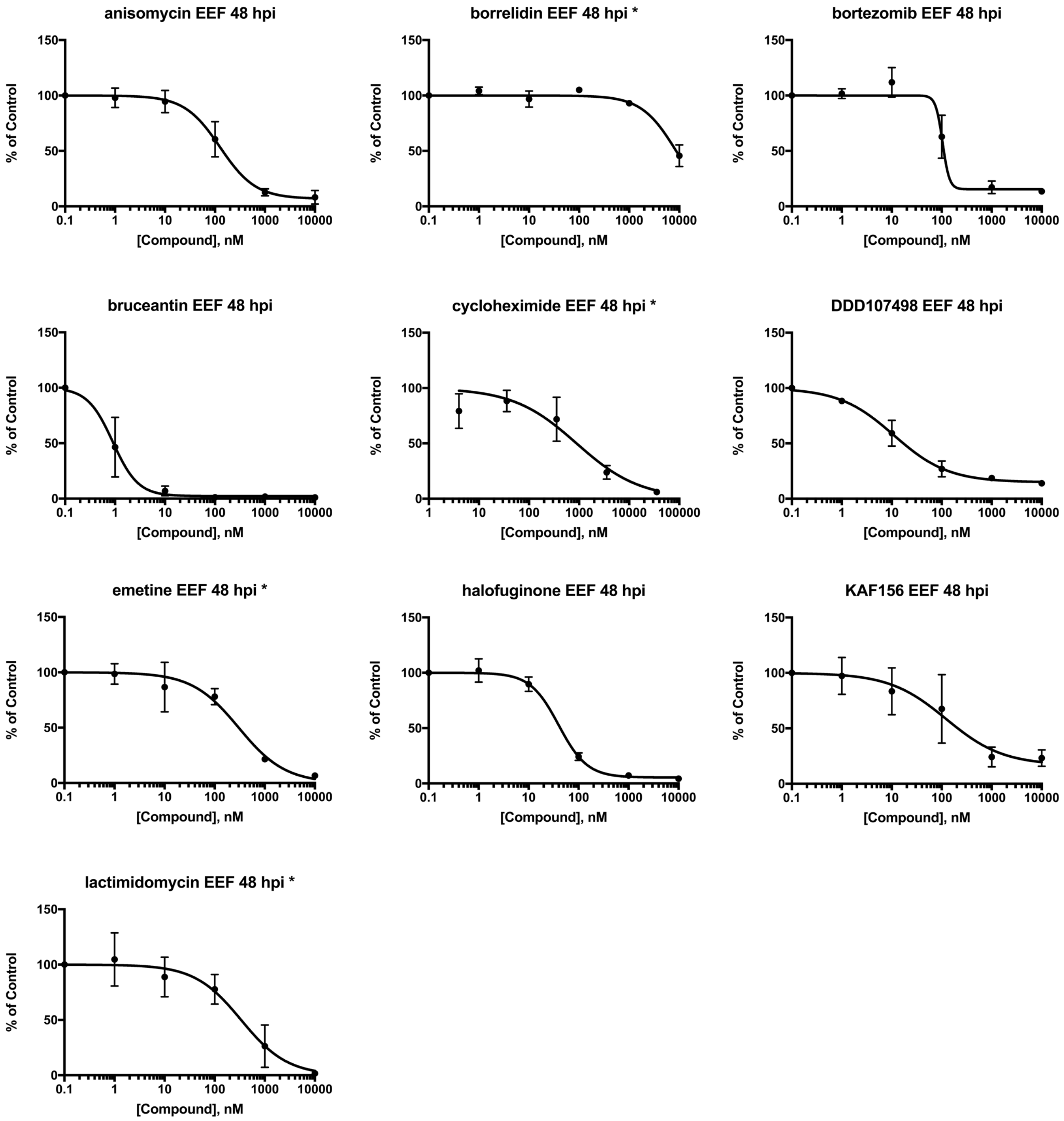
anisomycin EEF 48 hpi
borrelidin EEF 48 hpi *
bortezomib EEF 48 hpi
bruceantin EEF 48 hpi
cycloheximide EEF 48 hpi *
DDD107498 EEF 48 hpi
emetine EEF 48 hpi *
halofuginone EEF 48 hpi
KAF156 EEF 48 hpi
lactimidomycin EEF 48 hpi *
% of Control
0
50
100
150
[Compound], nM
0.1
1
10
100
1000
10000
100000

D

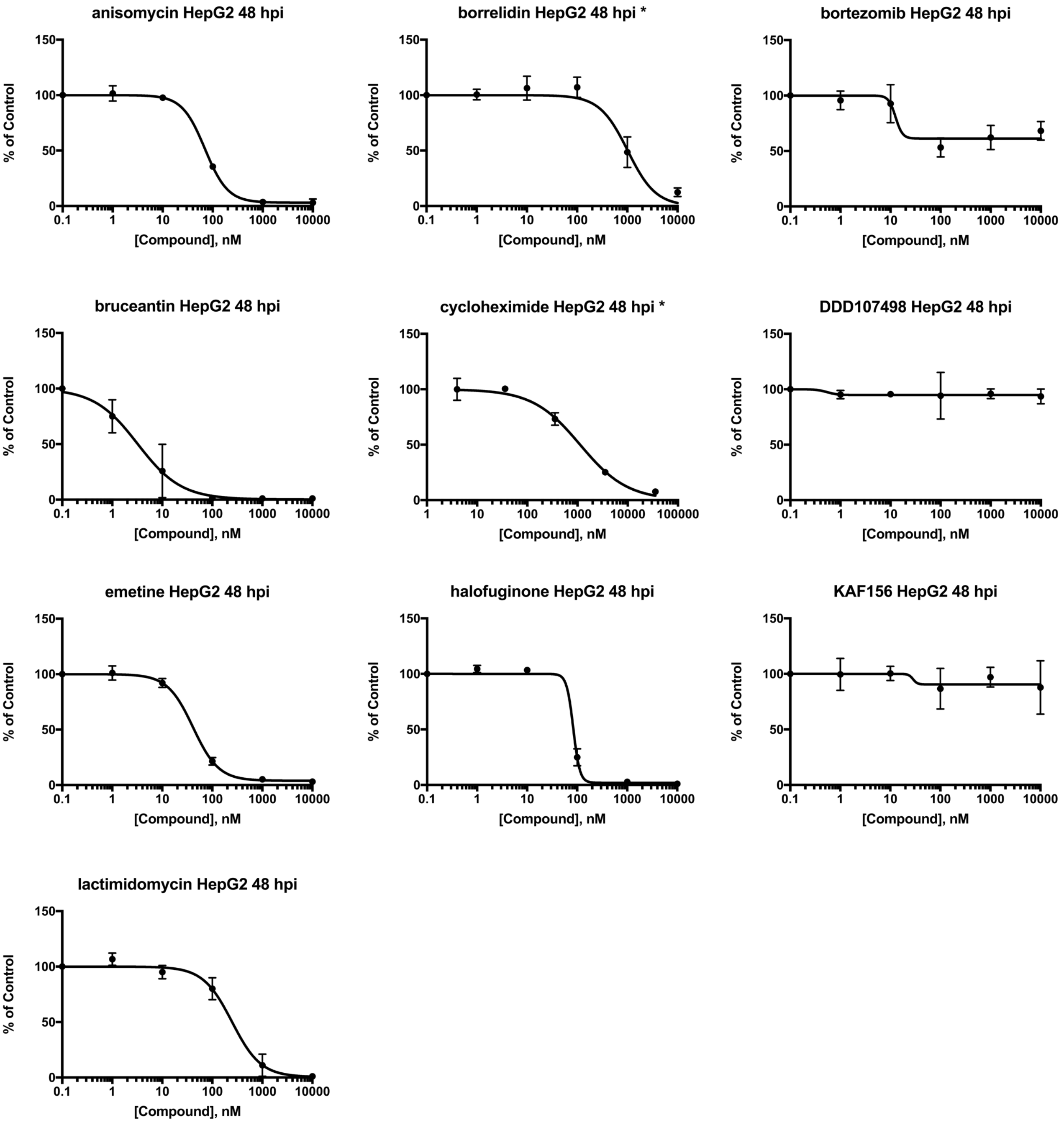

Figure S10. Estimating potency of translation inhibitors. Translation was quantified following acute pre-treatment in *P. berghei* liver stages during early schizogony (A) and matching in-image HepG2 cells (B), and in *P. berghei* liver stages during late schizogony (C) and corresponding in-image HepG2 cells (D), using 5-point, 10-fold serial dilution. Plots marked with an asterisk were fit with the bottom of the curve equal to 0, while all others were fit open, as detailed in Methods. Dataset as in Fig. 3 and Fig. S3-2; n ≥ 3 independent experiments.

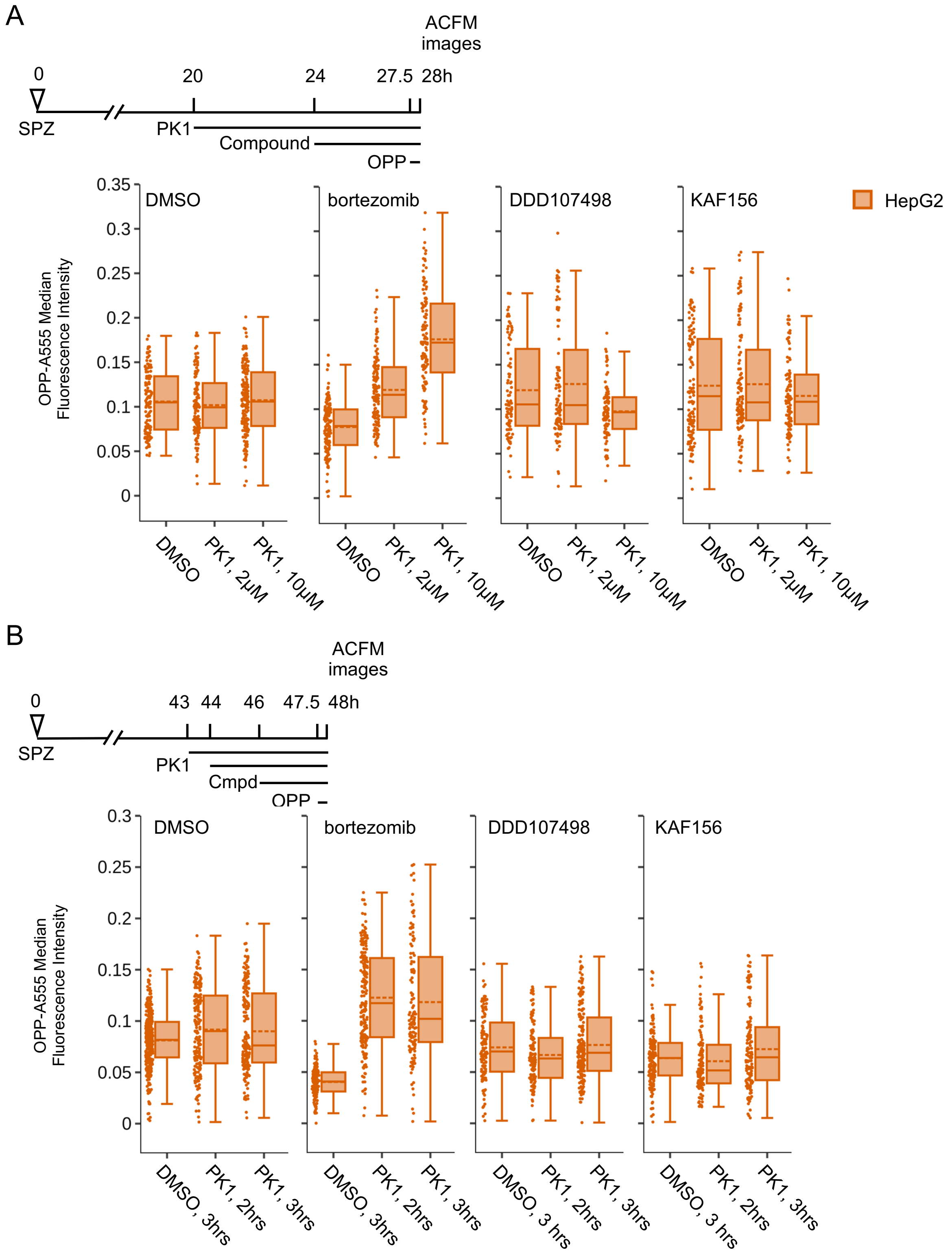

Figure S11. PK1 pre-treatment modulates bortezomib-induced changes in HepG2 protein synthesis. Experiment schematics and boxplots quantifying HepG2 translation after PK1 pre-treatment, then addition of DDD107498, KAF156, or bortezomib, as labeled. Each data point represents in-image HepG2 cells corresponding to the single parasite data quantified in Fig. 5A-B. [bortezomib] = 1 µM, [KAF156] = 0.5 µM, and [DDD107498] = 0.1 µM in A-B); [PK1] = labeled in A) and 20 µM in B). Boxplots show cumulative data from n=3 independent experiments, with mean additionally indicated by a dotted line.

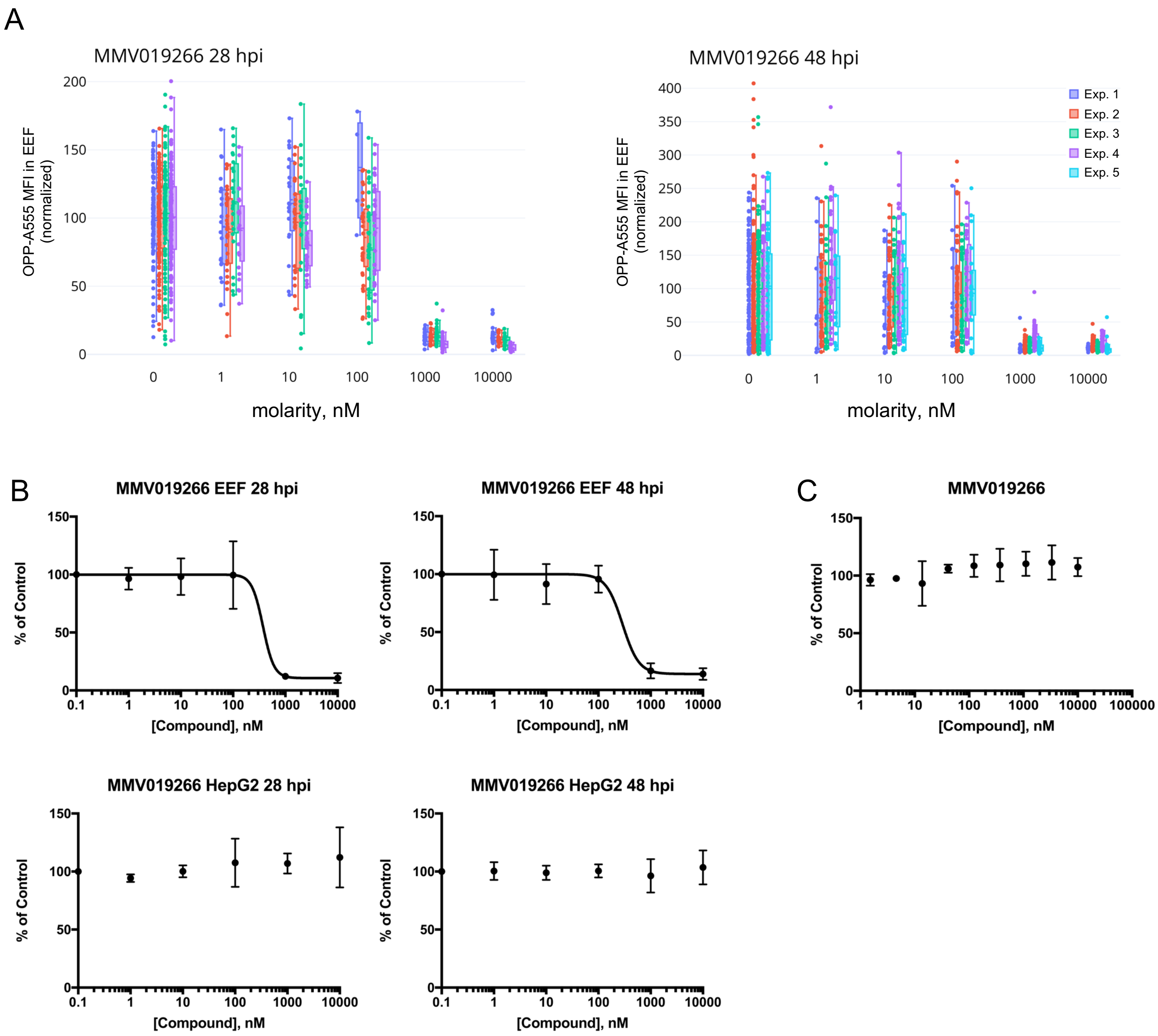

Figure S12. Concentration-response testing of MMV019266. Quantification of *P. berghei*-infected HepG2 translation following acute pre-treatment with MMV019266 in concentration response. A) Individual experiments shown for data summarized in Figure 6C, and fitted curves B) for both EEF and HepG2 translation inhibition. C) Fitted HepG2 cytotoxicity concentration response curve following treatment with 10-point, 3-fold serial dilution; maximal concentration 10µM in B-C). All data measured in n≥3 independent experiments.

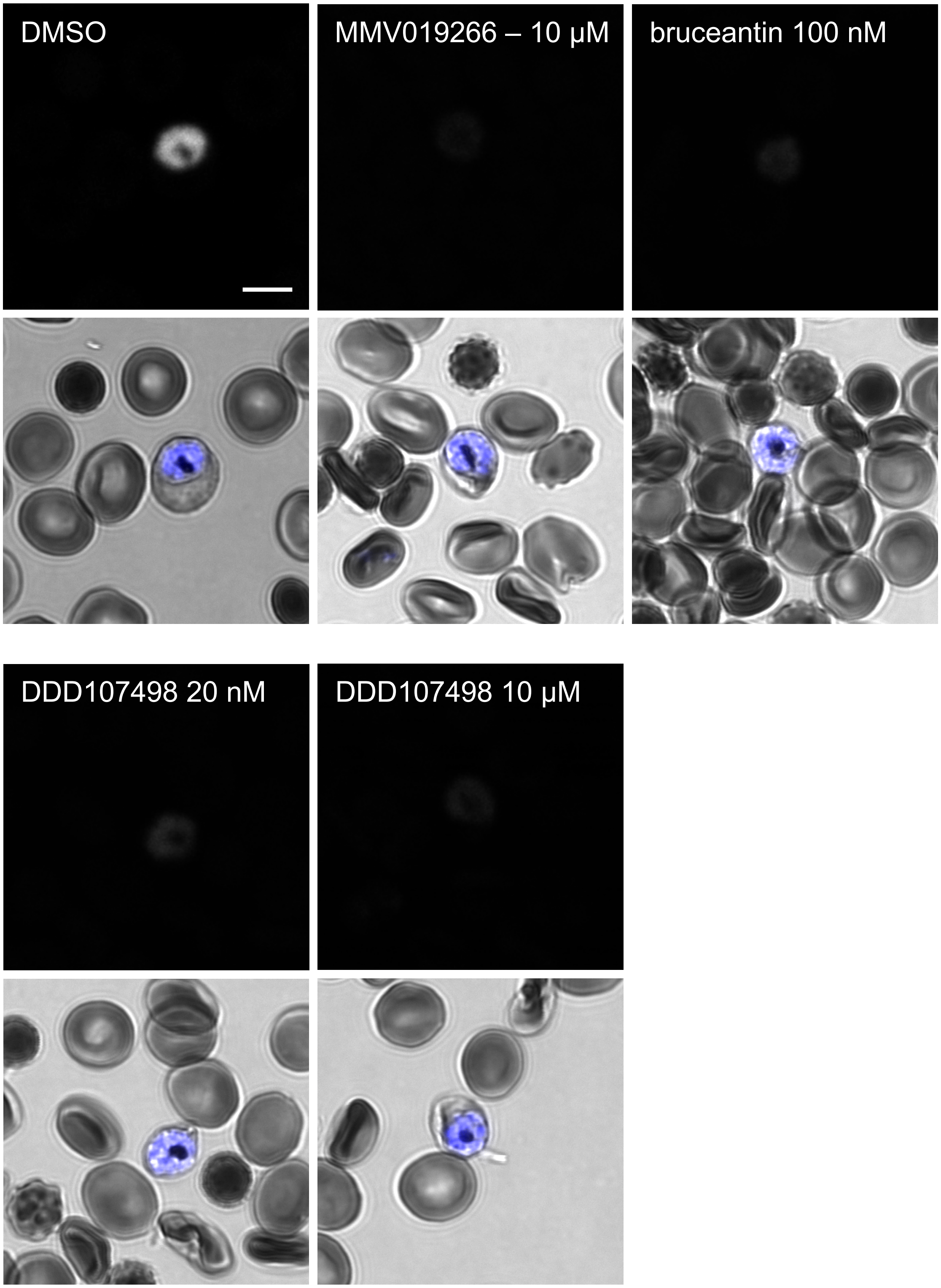

Figure S13. MMV019266 inhibits *P. falciparum* translation during blood stage schizogony. Representative single confocal images of *P. falciparum* blood stage schizonts following a 4h acute pre-treatment pulse of compound, as labelled for each image set. OPP-A555 labels the nascent proteome (grayscale), and brightfield images are merged with Hoechst-labeled parasite DNA (blue). All images were acquired with identical settings and are displayed at the same scale; scale bar = 5µm.
